# Comparative and evolutionary analysis of Arabidopsis RIN4-like/NOI proteins induced by herbivory

**DOI:** 10.1371/journal.pone.0270791

**Published:** 2022-09-27

**Authors:** Estefania Contreras, Manuel Martinez

**Affiliations:** 1 Centro de Biotecnología y Genómica de Plantas (CBGP), Instituto Nacional de Investigación y Tecnología Agraria y Alimentaria, Universidad Politécnica de Madrid, Madrid, Spain; 2 Departamento de Biotecnología-Biología Vegetal, Escuela Técnica Superior de Ingeniería Agronómica, Alimentaria y de Biosistemas, Universidad Politécnica de Madrid, Madrid, Spain; Universita degli Studi di Roma Tor Vergata, ITALY

## Abstract

The spider mite *Tetranychus urticae* is an economically important agricultural pest, which feeds on a broad spectrum of plant species. In an RNAseq experiment performed in our laboratory, 4 of the 15 members of the RIN4-like/NOI family of *Arabidopsis thaliana* were significantly overexpressed after *T*. *urticae* infestation. Two of them (NOI3 and NOI5) are shorter and harbour one NOI domain, which characterises this family, and the other two (NOI10 and NOI11) have two-NOI domains. The only member of this family characterized is RIN4, a two-NOI intrinsically disordered protein anchored to the plasma membrane and involved in plant defence against bacterial pathogens. The function of all other members of the RIN4-like/NOI *Arabidopsis* family and their putative role in herbivore defence remains unknown. We perform a comparative genomic analysis of RIN4-like/NOI sequences to study the evolutionary features of this protein family and the distribution of its members among species. We show that short one-NOI proteins were more numerous and exhibited lower disorder propensity compared to two-NOI members. NOI10 and NOI11, from the two-NOI group, are included in a clade-specific expansion of Brassicaceae with unique predicted posttranslational modification sites and clear predicted structural differences from RIN4. Our analysis suggests that the members of the RIN4-like/NOI family upregulated after mite feeding have novel functions different from those assigned to RIN4, likely involving adaptation to stress specialisation.

## Introduction

Understanding how plants signal and respond to pest attacks is essential to designing novel strategies for pest control. A model species among arthropod herbivores is the two-spotted spider mite *Tetranychus urticae*. In recent years, much effort has been made to decipher genes and signal transduction pathways in the model plant *Arabidopsis thaliana* involved in the response to the mite to eventually transfer this knowledge to crops [1]. In this context, recent comparative transcriptomic analyses have been revealed as robust tools to unravel plant responses to herbivore species [1, 2]. The results of these analyses highlighted the high number of receptors differentially regulated upon *T*. *urticae* infestation and the different combinations of receptor proteins induced upon specific herbivore attacks [2]. In addition to transcriptional regulation, the functionality of these receptors depends on their interactions with other proteins, which have not been analyzed in depth.

The induction of plant defences is initiated by activating receptors that recognize conserved molecular patterns or specific molecules of attackers in two ways. One involves the use of transmembrane pattern recognition receptors (PRRs) that respond to pathogen-, herbivore-, or damaged-associated molecular patterns (PAMPs, HAMPs, or DAMPs), triggering the pattern recognition receptor-triggered immunity (PTI). The other branch includes intracellular receptors that identify virulence molecules, known as effectors, activating the effector-triggered immunity (ETI) [3]. PTI and ETI can be mutually potentiated and are dependent on each other [4, 5]. Experimental and bioinformatic approaches pointed out that the *A*. *thaliana* RIN4 protein is an intrinsically disordered protein (IDP) under physiological conditions that regulates PTI and ETI [6, 7]. Intrinsic disorder plays a critical role in signaling and protein interaction networks in plants [8] and has been reported to be a key mechanism for sensing pathogen infections [9]. RIN4 homologues from different plant species and the NOI proteins from Arabidopsis are also IDPs [7]. Disordered regions provide versatility in interaction and undergo induced local folding upon binding to a range of molecules [7, 10, 11]. RIN4 possesses multiple putative sites for protein interaction in the disordered regions that consist in short semi-ordered regions named Molecular Recognition Features (MoRFs) [7]. A diversity of proteins involved in plant defense has been described as RIN4 interactors. Some of them are related to PTI, such as H^+^ATPases, which promote stomatal opening, or exocyst subunits implicated in callose secretion. Other RIN4 binding proteins such as Resistance to *Pseudomonas syringae pv*. *maculicola* 1 (RPM1) or Resistance to *Pseudomonas syringae* protein 2 (RSP2) are intracellular receptors with nucleotide binding-leucine-rich repeat (NBS-LRR) domains (NLRs). These receptors are resistance (R) proteins that mediate effector-triggered immunity (ETI) by recognising bacterial virulence effectors [12]. Multiple type III virulence effectors from the bacterial pathogen *Pseudomonas syringae* released to modulate plant host physiology and defense also target and modify RIN4. Posttranslational modifications of RIN4 mainly affect the regions containing MoRFs [13, 14]. Among the most studied perturbations of RIN4 are the phosphorylation of T166, which leads to the activation of RPM1 and thus to ETI, and the phosphorylation of S141 or S160, which enhances PTI. Other posttranslational modifications include proteolysis by the AvrRpt2 effector, ADP-ribosylation induced by AvrRpm1, or acetylation by HopZ3, HopZ5, and AvrBst [13, 15]. Similarly, host proteins also target RIN4 for different post-translational modifications, reinforcing the role of RIN4 as a hub in plant innate immunity [16].

RIN4 homologs have been identified in many plant species, indicating that RIN4 proteins in various species may fulfil similar roles [13]. All RIN4 proteins have two NOI (NO_3_-induced) domains located at the N- and C-terminal ends of the protein (N-NOI and C-NOI, respectively). The NOI domain was initially identified in a screening for nitrate-induced genes, although no link between NOI-containing proteins and nitrogen metabolism has been established [17]. RIN4 proteins also contain two or three conserved membrane-anchoring cysteine residues in the C-terminal region. These conserved motifs are sites of critical importance within RIN4. NOI-containing proteins, including RIN4, are grouped in the RIN4-like/NOI family. Besides RIN4, the *A*. *thaliana* genome harvests 14 RIN4-like/NOI proteins with unknown functions, named from NOI1 to NOI14 [6, 18]. Family members show limited conservation of primary sequences except for the presence of NOI domains and conserved C-terminal cysteine residues. Other than RIN4, only NOI10 and NOI11 contain both N- and C-NOI domains, and the other members are shorter and contain only C-NOI [7, 17].

Despite its relevance in plant defense, RIN4 has not been studied in the plant response to herbivory, and there is scarce information on the function of all other family members in *A*. *thaliana* and other species. With this in mind, the main objective of this work was to obtain novel findings on the role of NOI-containing proteins in plant defense. For that, a comparative genomic approach was performed to explore the evolutionary characteristics of the RIN4-like/NOI family and their distribution among species. This study was focused on the analysis of the *A*. *thaliana* NOI-containing proteins differentially expressed upon spider mite feeding to provide structural insights on their functionality.

## Results

### RIN4-like/NOI family proteins are differentially regulated in *A*. *thaliana* in response to herbivory

Exploring a comprehensive RNA sequencing analysis performed previously [1], four genes from the RIN4-like/NOI protein family (*NOI3*, *NOI5*, *NOI10*, and *NOI11*) were differentially expressed in *A*. *thaliana* rosettes after *T*. *urticae* infestation (Table 1). All were induced in the shorter time provided, 30 min of mite infestation. Searches were equally performed in the transcriptomic data used for a previous meta-analysis of *A*. *thaliana* challenged with the feeding of diverse arthropods [1]. The same four RIN4-like/NOI family members, *NOI3*, *NOI5*, *NOI10*, and *NOI11*, were found differentially regulated in several transcriptomic analyses using other arthropod species. However, the four genes were not induced simultaneously with any of the other arthropods (Table 2). Apart from these four genes, only the *NOI6* member was differentially regulated in any other analysis. Interestingly, *RIN4* was not induced or repressed by any herbivore stress. These results indicate the participation of a set of members of this protein family in the plant response to herbivore pests from a diversity of feeding modes.

**Table 1:** Expression of NOI genes in *A*. *thaliana* infested with *T*. *urticae*.

Gene name	Accession number	Time after infestation			
		30’	1h	3h	24h
*NOI1*	AT5G63270	ns	ns	ns	ns
*NOI2*	AT5G40645	ns	ns	ns	ns
*NOI3*	AT2G17660	3.66	2.65	2.43	ns
*NOI4*	AT5G55850	ns	ns	ns	ns
*NOI5*	AT3G48450	2.65	2.83	4.16	3.13
*NOI6*	AT5G64850	ns	ns	ns	ns
*NOI7*	AT5G09960	ns	ns	ns	ns
*NOI8*	AT5G18310	ns	ns	ns	ns
*NOI9*	AT5G48500	ns	ns	ns	ns
*NOI10*	AT5G48657	3.79	2.39	2.51	3.14
*NOI11*	AT3G07195	4.63	2.32	2.53	3.45
*NOI12*	AT2G04410	ns	ns	ns	ns
*NOI13*	AT4G35655	ns	ns	ns	ns
*NOI14*	AT5G19473	ns	ns	ns	ns
*RIN4*	AT3G25070	ns	ns	ns	ns

Values are expressed as Log2FC (Fold Change) compared to the expression in non-infested plants. ns values mean that the difference with the expression in non-infested *A*. *thaliana* plants was not statistically significant.

**Table 2:** Expression of NOI genes in *A*. *thaliana* infested with different arthropods.

Taxonomic group	Species and time after infestation	NOI 3	NOI 5	NOI 6	NOI10	NOI 11
Trombidiformes	*Brevipalpus yothersi 6d*	ns	ns	ns	ns	ns
	*Brevipalpus yothersi 6h*	ns	4.13	ns	1.09	ns
	*Brevipalpus yothersi 2d*	ns	4.13	ns	1.31	ns
Thysanoptera	*Frankiniella occidentalis 48h*	ns	ns	ns	ns	ns
	*Frankiniella occidentalis 24h*	ns	ns	ns	ns	ns
Diptera	*Liriomyza huidobrensis 4h*	ns	2.98	ns	ns	ns
Lepidoptera	*Pieris rapae 3h*	ns	3.37	ns	ns	ns
	*Pieris rapae 6h*	ns	2.10	1.36	ns	ns
	*Pieris rapae 12h*	2.63	5.00	ns	ns	ns
	*Pieris rapae 24h*	2.12	3.18	ns	ns	ns
	*Mamestra brassicae 2d*	ns	ns	ns	ns	ns
	*Pieris brassicae 2d*	-1.59	ns	-1.24	ns	-1.28
	*Spodoptera littoralis 8d*	ns	ns	ns	ns	ns
Hemiptera	*Myzus cerasi 3h*	ns	ns	ns	ns	ns
	*Myzus cerasi 6h*	1.64	ns	ns	ns	ns
	*Myzus cerasi 24h*	ns	ns	ns	ns	ns
	*Brevicoryne brassicae 72h*	ns	ns	ns	2.49	1.74
	*Ropalosyphum padi 3h*	ns	ns	ns	ns	ns
	*Ropalosyphum padi 6h*	1.27	ns	ns	ns	ns
	*Ropalosyphum padi 24h*	ns	ns	ns	ns	ns
	*Myzus persicae 3h*	ns	ns	ns	ns	ns
	*Myzus persicae 6h*	1.11	ns	ns	ns	ns
	*Myzus persicae 24h*	1.05	ns	ns	ns	ns
	*Bemisia tabaci 7d*	ns	ns	ns	ns	ns

Values are expressed as Log2FC (Fold Change) compared to the expression in non-infested plants. ns values mean that the difference with the expression in non-infested *A*. *thaliana* plants was not statistically significant.

### Distribution of RIN4-like/NOI family proteins in different species

Restricted regulation of a specific set of family members could be a consequence of evolutionary features. To investigate the distribution of this protein family, a sequence homology search for RIN4-like/NOI family proteins was conducted in databases of representative plant and algae species. The proteins obtained were classified into one or two NOI domain members (short one-NOI or long two-NOI) (Table 3). Members of the RIN4-like/NOI family were not found in green (Chlorophyta) and red (Rhodophyta) algae species. From mosses to flowering plants, a higher evolutionary expansion of short one-NOI members was observed compared to long two-NOI members. While a maximum of seven two-NOI members was found in *Glycine max*, up to 29 one-NOI members were present in the same species (Table 3).

**Table 3:** Number of one-NOI and two-NOI RIN4-like/NOI members in different species.

Taxonomic group	Species	Number of sequences	Number of long two-NOI sequences	Number of short one-NOI sequences
Rhodophyta	*Chondrus crispus*	0	0	0
	*Galdieria sulphuraria*	0	0	0
	*Cyanidioschyzon merolae*	0	0	0
Clorophyta	*Ostreococcus lucimarinus*	0	0	0
	*Chlamydomonas reinhardtii*	0	0	0
Embryophyta	*Physcomitrium patens*	9	5	4
	*Marchantia polymorpha*	0	0	0
Lycopodiophyta	*Selaginella moellendorffii*	6	0	6
Amborellales	*Amborella trichopoda*	6	1	5
Liliopsida	*Sorghum bicolor*	13	4	9
	*Brachypodium distachyon*	11	5	6
	*Hordeum vulgare*	22	3	19
Eudicotyledons	*Coffea canephora*	8	3	5
	*Nicotiana attenuata*	19	5	14
	*Solanum tuberosum*	13	3	10
	*Solanum lycopersicum*	14	5	9
	*Populus trichocarpa*	17	3	14
	*Medicago truncatula*	19	2	17
	*Glycine max*	36	7	29
	*Gossypium raimondii*	24	3	21
	*Theobroma cacao*	9	2	7
	*Brassica rapa*	25	4	21
	*Arabidopsis halleri*	14	3	11
	*Arabidopsis lyrata*	15	3	12
	*Arabidopsis thaliana*	15	3	12

**Fig 1:**
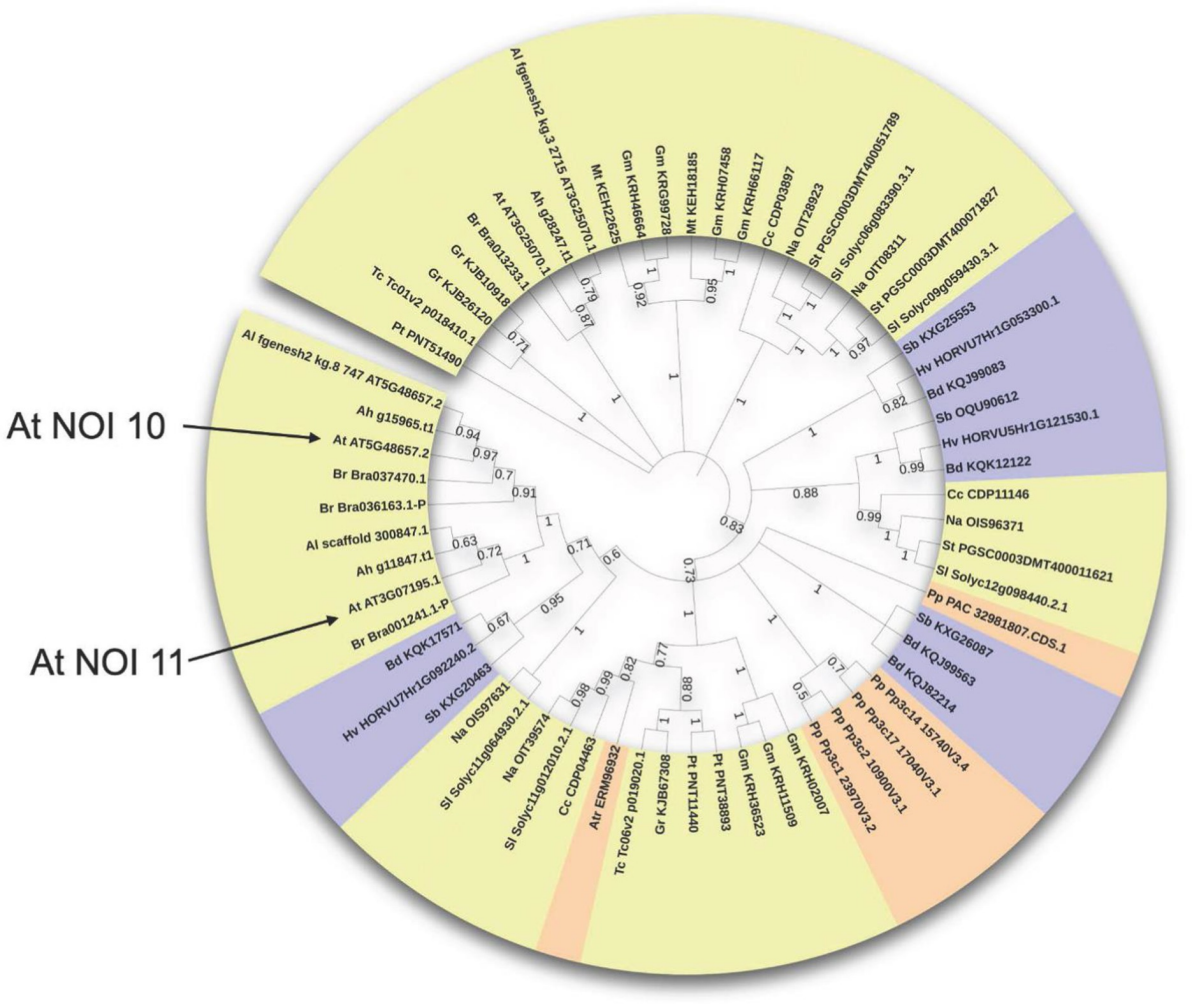
Phylogenetic tree of RIN4-like/NOI family long two-NOI proteins. Dicot labels are shown in yellow, monocot in purple, and PAS sequences in orange. The position in the tree of *A*. *thaliana* RIN4-like/NOI members upregulated after mite feeding is indicated. Branches are collapsed at 50% bootstrap, values higher than 50% are showed.

**Fig 2:**
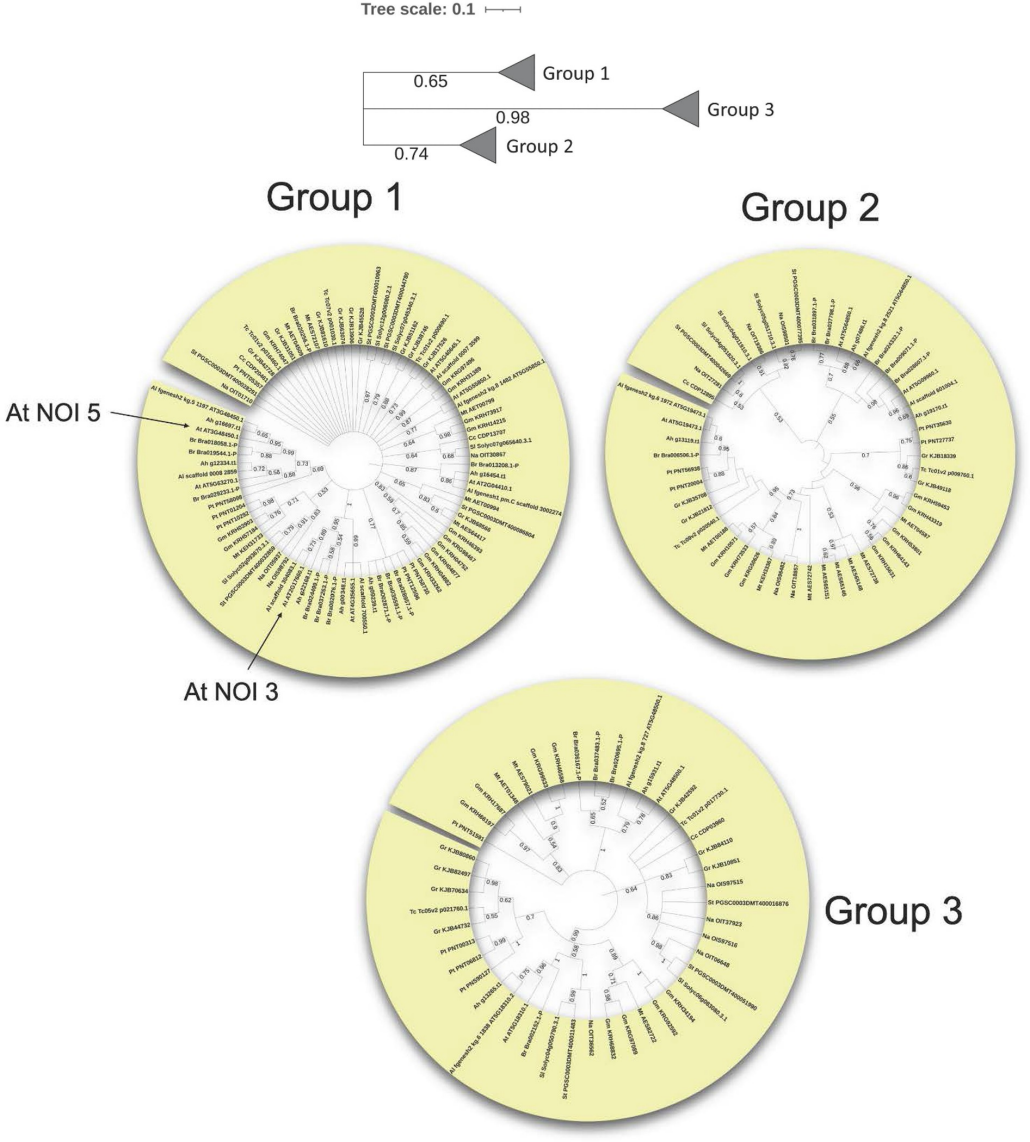
Phylogenetic trees of RIN4-like/NOI family short two-NOI proteins. A schematic tree for all dicot sequences and the individual group phylogenies are showed. The position in the tree of *A*. *thaliana* RIN4-like/NOI members upregulated after mite feeding is indicated. Bootstrap values are included.

A phylogenetic tree was constructed for the two-NOI RIN4-like/NOI members of all selected species (Fig 1). *A*. *thaliana* NOI10 and NOI11, up-regulated after mite feeding, grouped with other proteins from *Arabidopsis lyrata*, *Arabidopsis halleri*, and *Brassica rapa*, but did not group with *A*. *thaliana* RIN4 and the putative orthologs of RIN4 from other species. This finding suggests that these two-NOI group proteins belong to a clade-specific expansion of Brassicaceae. One-NOI members showed more complex phylogenetic relationships than two-NOI members. Separated trees were performed for monocots, dicots, and the PAS (*Physcomitrium*, *Amborella*, and *Selaginella*) group (Figs 2 and S1). One-NOI domains from dicots were divided into three phylogenetic groups (Fig 2). The first group contained *A*. *thaliana* NOI1, NOI2, NOI3, NOI4, NOI5, NOI12, and NOI13, the second group includes NOI6, NOI7, and NOI14, and the third group NOI8 and NOI9. Both one-NOI induced by mite feeding (NOI3 and NOI5) are included in the same group.

### Analysis of structural order-disorder propensity

Evolutionary RIN4-like/NOI expansions point to the diversification of structural properties. Former bioinformatics analyses on RIN4 homologs from different plant species and the NOI proteins from Arabidopsis pointed out the conservation of a platform of disorder and short-ordered NOI segments in the members of this protein family [7].

**Fig 3:**
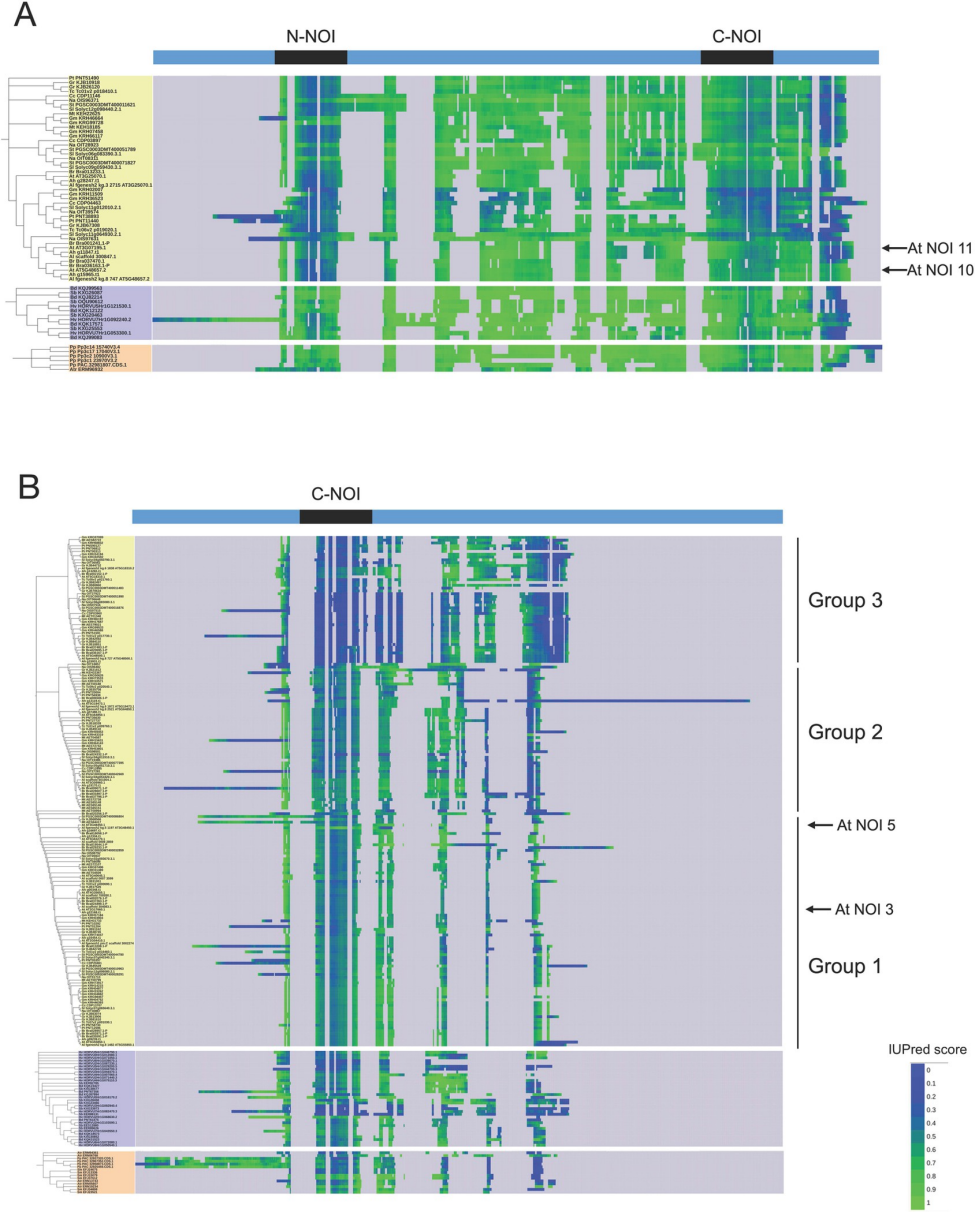
Disorder propensity of RIN4-like/NOI family proteins in different species. A) Long two-NOI sequences. B) Short one-NOI sequences. Disorder propensity is shown as IUPred score for each amino acid position between 0 and 1, where higher values correspond to a higher probability of disorder. Dicot labels are shown in yellow, monocot in purple, and PAS sequences in orange. Phylogenetic groups in short one-NOI dicot members and the sequences of *A*. *thaliana* RIN4-like/NOI members upregulated after mite feeding are indicated.

To extend the analysis to the full set of members analyzed in this work and investigate the structural evolution of proteins in the RIN4/NOI family, we analyzed the order-disorder propensity of each amino acid position in the sequences using the IUPred tool (S1 Dataset). Disorder propensity scores were plotted for each position as a color gradient in a multiple sequence alignment. The location of each taxon in the alignment corresponds to its location in the phylogenetic trees built separately for dicot, monocot, and PAS sequences (Fig 3A).

As expected, a high disorder propensity was observed along with all two-NOI sequences (Fig 3A), except in the conserved NOI domain regions, especially in the N-NOI from dicots. Besides, lower disorder propensity scores were observed in two-NOI sequences from dicots compared to those from monocots and *Physcomitrium*. The only two-NOI *Amborella* sequence showed a disorder propensity comparable to the scores observed in two-NOI dicot sequences to which it is closely related (Fig 3A). NOI10 and NOI11 from *A*. *thaliana* exhibited an average pattern of disorder propensity scores along the sequence.

The one-NOI sequences showed an overall low disorder propensity, mainly due to the lack of the intrinsically disordered region between the N-NOI and C-NOI domains, with the dicot sequences from group 3 having the lowest disorder propensity (Fig 3B). Dicot sequences from group 1, containing NOI3 and NOI5, have the highest disorder propensity scores, especially around the conserved C-terminal cysteine.

### Evolution of predicted phosphorylation sites

**Fig 4:**
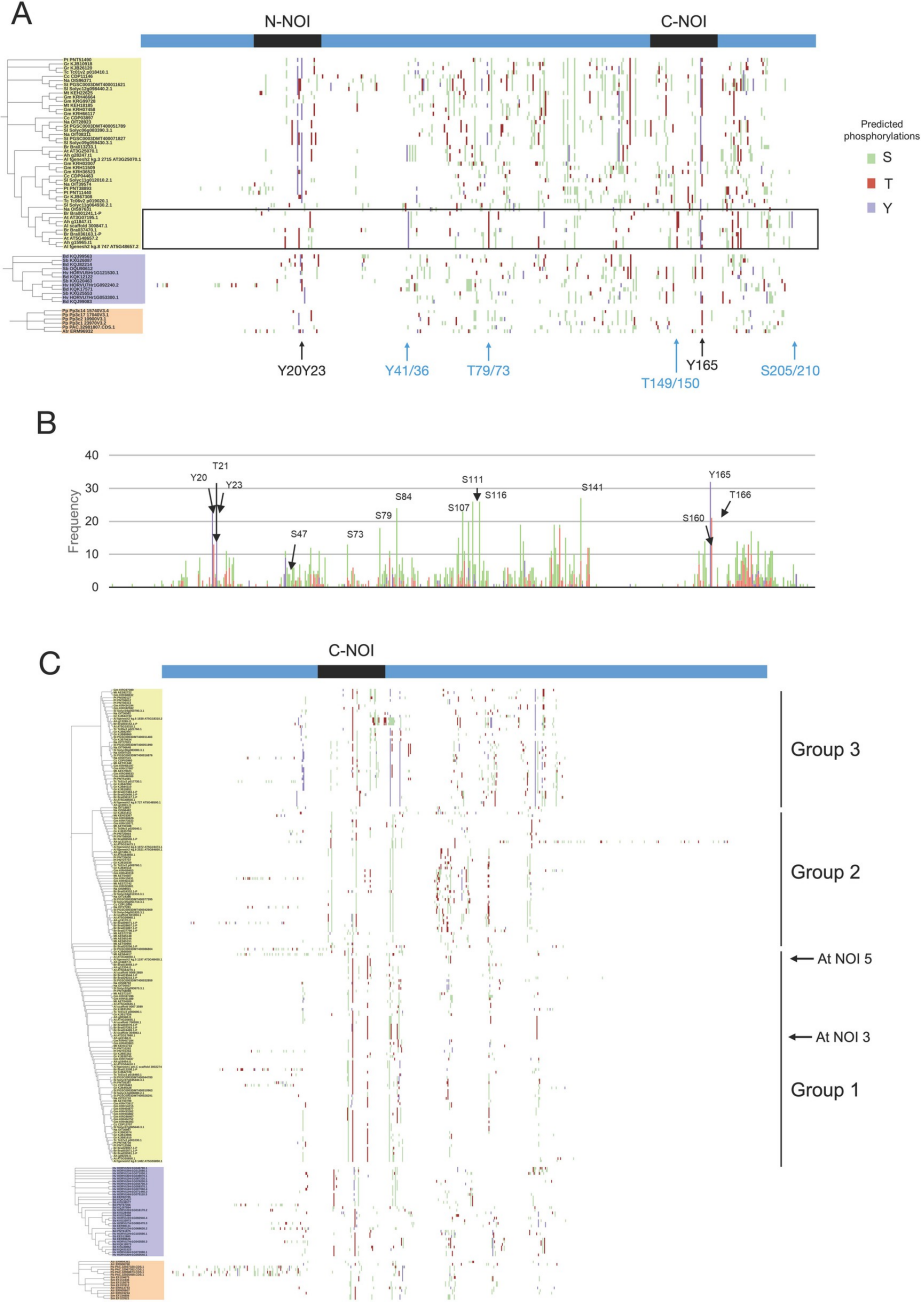
Predicted modification sites of RIN4-like/NOI family proteins in different species. A) Long two-NOI sequences. The group of sequences containing NOI10 and NOI11 is marked with a rectangle. The locations of relevant conserved modifications in this group are shown in blue. B) Recurrence of the predicted modifications per site in long two-NOI dicot sequences, determined as the number of sequences that contains a specific modification. B) Short one-NOI sequences. In A) and B), dicot labels are shown in yellow, monocot in purple, and PAS sequences in orange. Phylogenetic groups of short one-NOI dicot members and the sequences of *A*. *thaliana* RIN4-like/NOI members upregulated after mite feeding are indicated.

Structural divergences could be associated with changes in post-translational modification sites. To evaluate this aspect, phosphorylation of serine, threonine, and tyrosine residues was predicted for all sequences and was depicted in multiple sequence alignments (Fig 4 and S1 Dataset). Again, phylogenetic trees built separately from dicot, monocot, and PAS sequences were added to the alignment matching the sequence of each taxon.

Some of the predicted modifications exhibited a high degree of conservation in the two-NOI sequences (Fig 4A). Examples are one or two tyrosine residues located in the N-NOI (in RIN4, Y20, and Y23) and the tyrosine in the C-NOI (Y165) implicated in the activation of RPM1 [19], as well as the serine residues S111, S116, or S141, the latter known to be phosphorylated by the flg22 peptide from bacterial flagellin [20]. The group containing NOI10 and NOI11 displayed a predicted modification pattern that differed from other sequences. This group lacks some of the well-conserved modifications, such as Y20, Y23, and S111, and also S160, which is relevant for PTI. Instead, some modifications well conserved in the NOI10/NOI11 group are unique, such as Y41/Y36, T79/T73, T149/T150, or S205/S210. According to these divergences in amino acid sequences, relevant experimentally determined RIN4 modifications, such as T21, S160, or T166 [21, 22], showed a lower degree of conservation in the dicot sequences (Fig 4B).

Some potentially phosphorylated residues were also found in the one-NOI sequences, with variable conservation among the phylogenetic clades (Fig 4C). Marked differential patterns appeared between each of the three dicot groups, the monocot, and the PAS clades. A comparison between the C-NOI domains of the long and short NOI proteins indicated that the well-conserved Y165 of the two-NOI sequences is not present in the one-NOI and that the S160 and T166 residues are mostly present in the sequences belonging to the dicot group 1 containing the NOI3 and NOI5 proteins.

### Amino acid evolution rate of dicot RIN4-like/NOI family proteins

**Fig 5:**
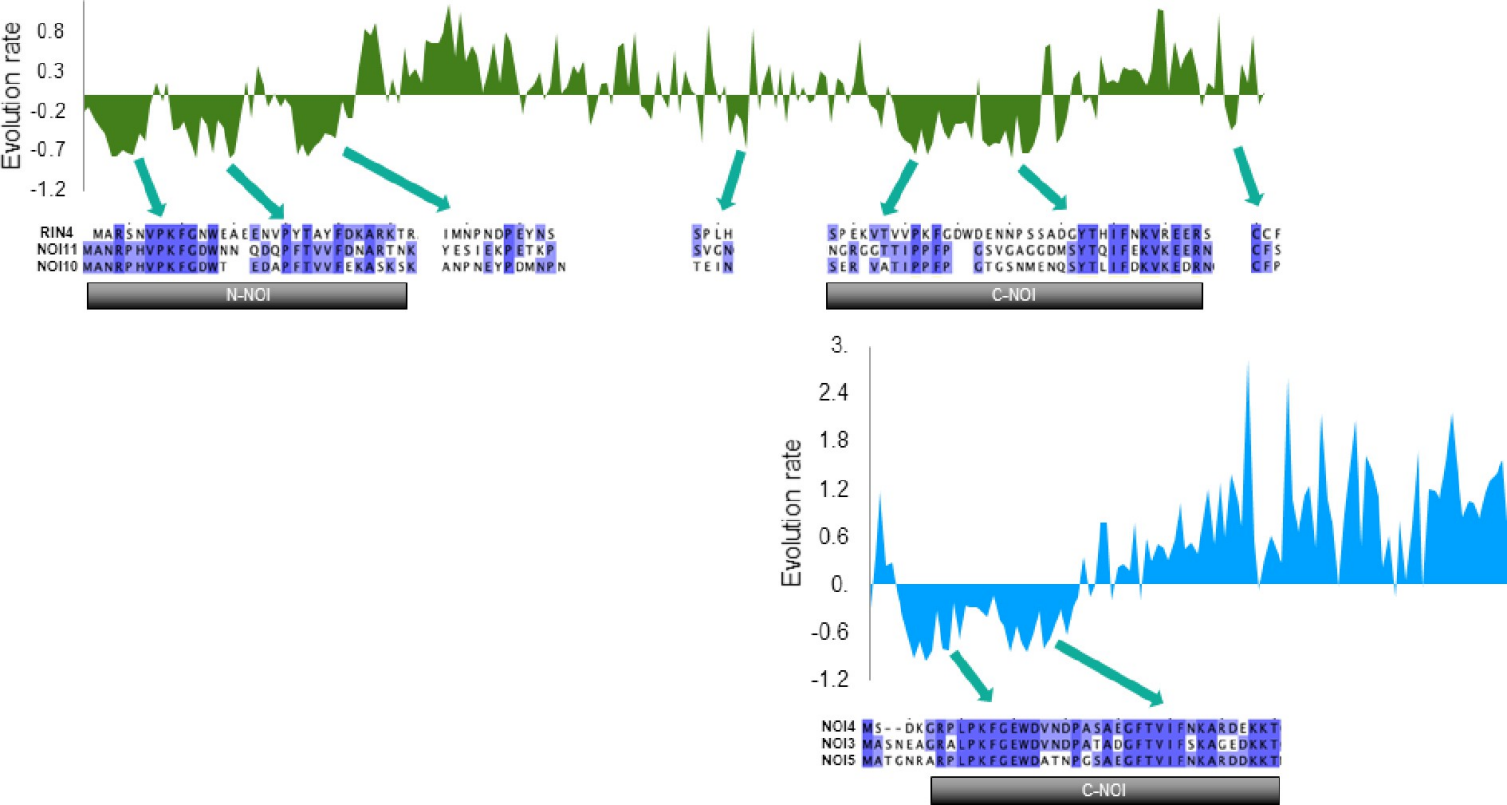
Amino acid evolution. Rate per site for long two-NOI sequences (dark green) and short one-NOI sequences (light blue) from dicot species. Partial alignments of the reference sequences, RIN4 for long two-NOI and NOI4 for short one-NOI, with the mite-induced members are shown below the negative evolution rates.

Changes in phosphorylation patterns are closely related to amino acid evolution. To investigate whether the evolution of the RIN4-like/NOI family proteins is different in distinct regions, we determined the rate of amino acid substitution in each position using all dicot sequences. RIN4 and NOI4 were selected as reference sequences of two-NOI and one-NOI proteins (Fig 5). Negative evolution rates were found mainly in the conserved NOI domains of both one- and two-NOI, where fewer amino acid substitutions than those produced by chance (evolution rate zero) are expected. In contrast, positive substitution rates were observed in the rest of the sequence, defined by a higher intrinsic disorder. Higher rates of positive amino acid substitution were found for the one-NOI sequences, highlighting the greater diversity of these proteins.

The reference sequences and the NOI members upregulated after mite infestation were compared in the regions showing a negative evolution rate. The one-NOI reference sequence and the NOI3 and NOI5 members showed high conservation in the C-NOI domain, the unique region with a negative evolution rate. In contrast, the NOI10 and NOI11 sequences displayed many amino acid changes with the reference sequence in the regions where the two-NOI dicot proteins have low substitution rates (Fig 5). Remarkably, we also observed that the N-NOI domains of NOI10 and NOI11 were more conserved and had lower amino acid substitution rates than their corresponding C-NOI domains.

### Structural features of the C-NOI of *A*. *thaliana* members

**Fig 6:**
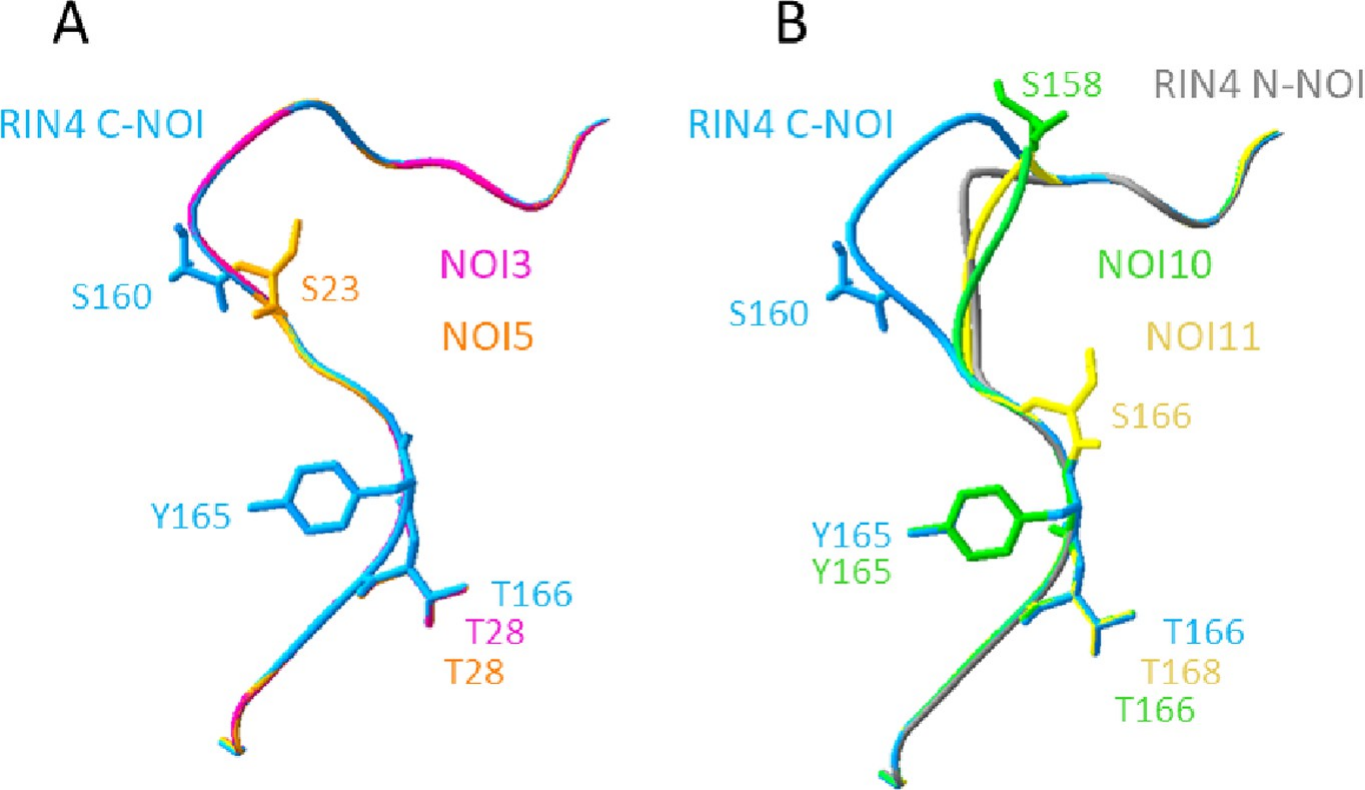
Structural features of the C-NOI domains of *A*. *thaliana* RIN4-like/NOI members. A) Comparison of RIN4 and the predicted structures of NOI3 and NOI5. B) Comparison of RIN4 and the predicted structures of NOI10, NOI11, and the N-NOI domain of RIN4. Predicted phosphorylated amino acids are shown in sticks.

Since the C-NOI domains of the mite-induced members showed variable conservation, predictions of the C-NOI structure for these proteins were performed to detect any sequence-structure relationship. The solved structure of the RIN4 C-NOI peptide was used as a template. While the predicted structures of the C-NOI domains of NOI3 and NOI5 were highly similar to those of RIN4, the C-NOI domains of NOI10 and NOI11 showed clear structural differences (Fig 6).

The solved structure of RIN4 showed an ENNPS_156-160_ motif, characterized by a polar glutamic acid residue and a proline, which promotes a loop turn structure connecting a short alpha-helix (GDWDE_152-156_) and a beta-sheet (HIFN_167-170_) (Fig 6A). This loop favors an exposed location of the potentially phosphorylated residue S160 of RIN4 and is maintained in NOI3 and NOI5 (Fig 6A). On the contrary, the predicted structures of NOI10 and NOI11, and also the N-NOI of RIN4, showed a shorter loop with potentially phosphorylated serine residues distantly located from RIN4 S160 (Fig 6B). In contrast, the short Y/FTxxF beta-sheet motif containing the phosphorylated T166 of RIN4 was structurally conserved in the three two-NOI proteins and in NOI3 and NOI5.

### Analysis of binding regions

**Fig 7:**
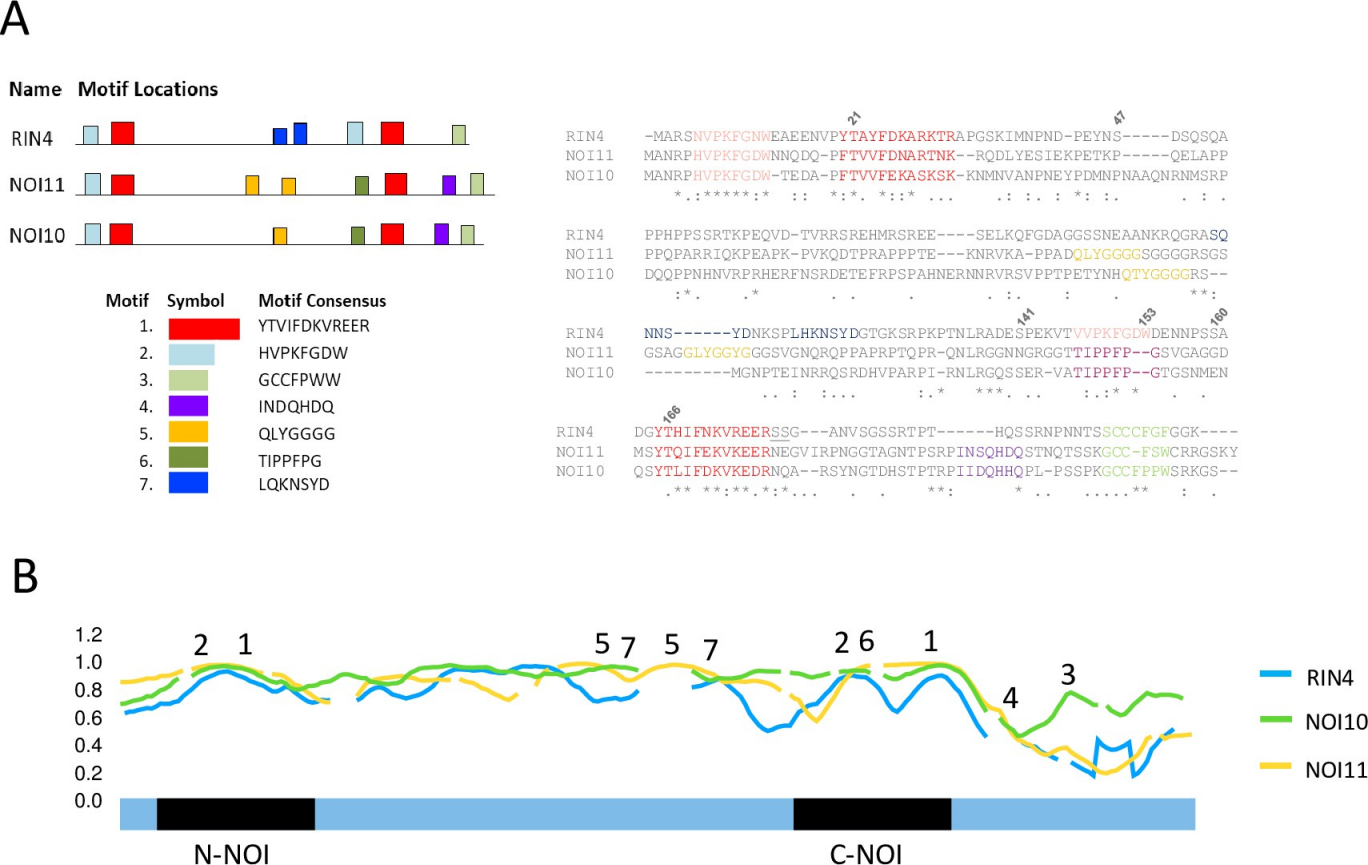
Analysis of binding regions. A) Scheme (left) and alignment (right) showing the location of the conserved motifs specific for *A*. *thaliana* NOI10/NOI11 or shared with RIN4. B) Disorder binding propensity of *A*. *thaliana* RIN4, NOI10, and NOI11 amino acids shown as Anchor scores. Numbers mark the approximate location of the conserved motifs described in A).

In addition to modifications in the conserved NOI domains, other motifs could be differentially present in the RIN4, NOI10, and NOI11 sequences. Using the MEME tool with a highly restrictive approach, we found some shared or specific motifs for the NOI10/NOI11 and RIN4 sequences (Fig 7A). Apart from the conserved motifs in the N-NOI, C-NOI, and C-terminal regions, three motifs were present only in NOI10/NOI11 or in RIN4.

To check if these motifs are located in disordered regions differing in their binding capacity, the disordered binding propensity for each amino acid was predicted for RIN4, NOI10, and NOI11 by Anchor2 server (Fig 7B). The three proteins showed high Anchor2 scores along with the sequences, including the N- and C-NOI regions, revealing that a large extent of the sequence is likely to gain stabilizing energy by interacting with protein interactors. However, some regions along the three sequences had distinct disordered binding propensities. Interestingly, one of the specific NOI10/NOI11 motifs, the QLYGGGG motif, was placed in NOI10 and NOI11 regions with higher disordered binding region scores than in RIN4. Therefore, the local sequence-structure differences observed in NOI10 and NOI11 compared to RIN4 were consistent with a differential pattern of interacting partners.

## Discussion

The Arabidopsis RIN4 protein has been extensively studied as a key player in plant defense against biotic stressors. Its intrinsically disordered structure, especially the region between the conserved N-NOI and C-NOI domains, gains stability by binding to a diversity of proteins involved in immunity. Although IDPs are common targets for pathogen effectors [23], it has been hypothesized that IDPs can evolve more rapidly due to their structural flexibility, allowing the plant to follow the evolutionary pace of pathogens while maintaining the motifs required to retain essential functions [7]. Therefore, this structural feature places RIN4 in a central position in the signaling and defense transduction pathways [13]. Whereas *A*. *thaliana* RIN4 and well-conserved putative RIN4 orthologs in tomato, Nicotiana, soybean, and lettuce have been associated with plant defense [24–26], the additional 14 RIN4-like/NOI members present in *A*. *thaliana* have been scarcely studied. Interestingly, *T*. *urticae* feeding induced upregulation of four of them, NOI3, NOI5, NOI10, and NOI11, but not of RIN4. As these members of the NOI family are also disordered proteins, some functionality similar to that of RIN4 may be hypothesized. However, pronounced structural differences should contribute to variations in the role of members with one or two NOI domains.

NOI3 and NOI5 are one-NOI members, which are shorter and miss the intrinsically disordered scaffold region between the two NOI domains. As a consequence, the space for multiple protein-protein interactions is drastically reduced, and the scaffold function conferred to RIN4 is largely lost. In Arabidopsis, several short one-NOI members were detected in transcriptomic assays as differentially regulated after a wide range of biotic and abiotic stresses, such as viruses, fungi, heavy metals, or genotoxic stress [27–30] supporting a role in plant defense, detoxification, or homeostasis. Interestingly, one-NOI members showed higher expansion and evolution rate scores compared to two-NOI proteins, suggesting that one-NOI members expanded to adapt and specialize to different stresses. In fact, of the 12 one-NOI members, only the expression of phylogenetically related NOI3 and NOI5 was induced after mite feeding. Likewise, only NOI3 and NOI5, and in fewer cases also NOI6, were differentially regulated in *A*. *thaliana* in response to the feeding of diverse phytophagous pests. All of these data suggest that short proteins in the RIN4/NOI family could have a marked specialization, with NOI3 and NOI5 being the most relevant in response to arthropod feeding.

It is notable that two *A*. *thaliana* long two-NOI proteins, NOI10 and NOI11, but not RIN4, were induced after mite feeding. Although the NOI domains are the most conserved regions of the protein, showing a low evolution rate, N-NOI and C-NOI appear to have evolved independently of each other [31]. NOI10 and NOI11 shared important differences with RIN4 affecting predicted posttranslational modification sites relevant to the functionality of RIN4 and the structure of the C-NOI domains. Most of these sites resides within the MoRFs motifs previously identified [7]. The phosphorylation of T166 in RIN4 is crucial for RPM1 triggering of defence responses against pathogenic bacteria. This RPM1-mediated ETI is enhanced by phosphorylation of T21 and S160 [22] and antagonizes S141 phosphorylation, suppressing PTI responses triggered by flagellin-mediated modification of S141 [20]. Whereas the predicted phosphorylation sites T21 and T166 are well conserved in almost all analyzed sequences, including NOI10 and NOI11, the S160 residue in the C-NOI domain was not present in NOI10 and NOI11. Otherwise, S141 was present in NOI10 but not in NOI11. Furthermore, ADP-ribosylation of D153 is necessary for complete phosphorylation of T166 in RIN4 [32]. This residue is also absent in the sequences of NOI10 and NOI11. All of these data suggest that NOI10/NOI11 do not share a common pattern with RIN4 in response to PTI elicitors, such as flagellin, or bacterial effectors. Supporting this hypothesis, while the AvrRpt2 cleavage site in N-NOI is present in the NOI10/NOI11 group as in RIN4, the second AvrRpt2 cleavage site in C-NOI was not conserved in NOI10/NOI11. It has been proposed that RIN4 could function as a decoy that mimics the actual effector target, then reduce pathogen virulence [33]. As NOI10/NOI11 lacked many of the well-conserved modifications or cleavage sites of RIN4 triggered or produced by bacterial effectors, these proteins could probably not play this RIN4-suggested role. Furthermore, three-dimensional predictions showed a differential exposition of key residues in the C-NOI. The specific S160 residue for RIN4 is located in a higher loop connecting the β-sheet following T166 and the α-helix harbouring D153, which is substituted at this position by a phosphorylating S or T residue in NOI10/NOI11. Collectively, these findings support the relevance of the C-NOI domain in the specific response of NOI proteins to various biotic stresses.

Additional signals come from the structure of long two-NOI proteins. Intrinsic disorder provides a versatile protein binding region between the N-NOI and C-NOI domains. As disorder propensity scores were slightly lower when comparing long two-NOI dicot with monocot and PAS sequences, functional specialization along evolution may be hypothesized for the two-NOI long members, as suggested for one-NOI short members. Specifically, NOI10/NOI11 are included in a clade expansion of Brassicaceae where differential motifs were discovered, a fact in line with this hypothesis. In addition, tolerance of intrinsically disordered regions to mutations has been related to the ability of some protein families to expand and gain more specific functions over time [34]. Numerous examples of signalling hub families, such as the transcription factors of the TCP and DOF families, show an overall disordered structure in the regions that do not bind DNA with high variation in their amino acid sequences according to a diversification of their potential interactors [35, 36]. Therefore, specialization could likely be associated with variations in the amino acid sequence that change the kind of proteins that can be recruited by each two-NOI member.

All these results are in agreement with the hypothesis of diversification along with evolution for these proteins caused by evolutionary forces that shape their interactions with both pathogen effectors and endogenous protein partners [7], The lack of members in algae and the liverwort *M*. *polymorpha* correlates with the assumption that many actors of the plant immune system appeared during land colonization. In this process, novel threats caused by pathogens and phytophagous organisms underwent dramatic expansions in several immune-related families [37]. An example is soybean, which has four RIN4 homologs, and some of them acquired the ability for R protein interaction and ETI initiation but lost the function for PTI mediation [33]. This hypothesis might also be true for proteins of the *A*. *thaliana* RIN4-like family such as NOI10 or NOI11. These proteins could have specialized in sensing a variety of new elicitors instead of bacterial effectors and could become guardees of some NB-LRR proteins under specific stresses such as arthropod feeding.

## Conclusions

Collectively, our results point to an expansion and diversification of the RIN4-like/NOI family to optimise the response of the plant to diverse biotic stresses. The evolutionary and structural features of the four *A*. *thaliana* mite-induced RIN4-like/NOI members support this conclusion. In particular, the two-NOI proteins are good examples, as relevant differences are found when mite-induced NOI10 and NOI11 proteins are compared with RIN4. Variations in the conservation of posttranslationally modified residues, the structure of the C-NOI domain, and the predicted binding motifs in the disordered regions strongly suggest specialization during evolution. These changes would likely be associated with distinct binding of elicitors and recruiting proteins leading to specific responses to particular biotic stresses.

## Methods

### Sequence selection

RIN4-like protein sequences from selected species were retrieved from Ensembl Plants release 51 database (https://plants.ensembl.org/), using the following assemblies: *Amborella trichopoda*, AMTR1.0; *Arabidopsis halleri*, Ahal2.2; *Arabidopsis lyrata*, v.1.0; *Arabidopsis thaliana*, TAIR10; *Brachypodium distachyon*, Brachypodium_distachyon_v3.0; *Brassica rapa*, Brapa_1.0; *Coffea canephora*, AUK_PRJEB4211_v1; *Glycine max*, Glycine_max_v2.1; *Gossypium raimondii*, Graimondii2_0_v6; *Hordeum vulgare*, IBSC_v2; *Medicago truncatula*, MedtrA17_4.0; *Nicotiana attenuata*, NIATTr2; *Physcomitrium patens*, Phypa_V3; *Populus trichocarpa*, Pop_tri_v3; *Selaginella moellendorffii*, v1.0; *Solanum lycopersicum*, SL3.0; *Solanum tuberosum*, SolTub_3.0; *Sorghum bicolor*, Sorghum_bicolor_NCBIv3; *Theobroma cacao*, Theobroma_cacao_20110822. *A*. *thaliana* RIN4 (AT3G25070) and RIN4-like proteins AT5G63270 (NOI1), AT5G40645 (NOI2), AT2G17660 (NOI3), AT5G55850 (NOI4), AT3G48450 (NOI5), AT5G64850 (NOI6), AT5G09960 (NOI7), AT5G18310 (NOI8), AT5G48500 (NOI9), AT5G48657 (NOI10), AT3G07195 (NOI11), AT2G04410 (NOI12), AT4G35655 (NOI13) were used as a reference in blastp (protein-protein BLAST) tool to search against the protein database of each species with a search sensitivity of distant homologies. The sequences found in each species were used as a reference to make new searches against the same species database. All sequences found were compared and those without NOI domain were removed. Only one isoform for each selected protein was used to minimise redundancy. Sequences are compiled in S2 Dataset.

### Phylogenetic analysis

Alignments of the amino acid sequences were performed using the default parameters of MUSCLE in MEGA11 software [38]. Phylogenetic trees were then constructed by maximum likelihood method with Jones-Taylor-Thornton amino acid substitution model and a BIONJ starting tree. All positions with less than 70% site coverage were eliminated. Branch support was determined by the bootstrap method (1000 replications). Abbreviations used: Atr, *A*. *trichopoda*; Ah, *A*. *halleri*; Al, *A*. *lyrata*; At, *A*. *thaliana*; Bd, *B*. *distachyon*; Br, *B*. *rapa*; Cc, *C*. *canephora*; Gm, *G*. *max*; Gr, *G*. *raimondii*; Hv, *H*. *vulgare*; Mt, *M*. *truncatula*; Na, *N*. *attenuata*; Pp, *P*. *patens*; Pt, *P*. *trichocarpa*; Sm, *S*. *moellendorffii*; Sl, *S*. *lycopersicum*; St, *S*. *tuberosum*; Sb, *S*. *bicolor*; Tc, *T*. *cacao*.

### Prediction of structure and sequence features

The disordered regions of all sequences were predicted using IUPred2 [39]. For the prediction of disordered binding regions, ANCHOR2 tool was used [39] and serine, threonine, or tyrosine phosphorylation sites were predicted using NetPhos 3.1 server [40]. The probabilities of the residues being part of a disordered region (range 0–1) and phosphorylation sites were represented as heatmap datasets from a multiple sequence alignment of the sequences and the corresponding phylogenetic tree using iTOL [41]. Conserved motifs in RIN4, NOI10, and NOI11 were determined using the MEME tool in the MEME suite 5.3.3 [42] with classic mode and zero or one occurrence per sequence.

### Amino acid evolution rate

The rates of evolution of each amino acid position were estimated by the empirical Bayesian inference method with the Jones, Taylor, and Thornton amino acid substitution model using Rate4Site [43]. Multiple sequence alignments of two-domain and one-domain NOI sequences from dicots and default starting trees computed by the software were used as input. *A*. *thaliana* RIN4 and NOI4 were used as reference sequences for each group. Branch lengths were optimised using a gamma model.

### Structure modelling

The structures of the NOI domain of NOI3, NOI5, NOI10, and NOI11 were predicted using Expasy SWISS-MODEL [44] based on homology modelling with the solved structure of a RIN4 peptide fragment complexed with the AvrB effector protein from *Pseudomonas savastanai* (2nud.1.B). This fragment contains the C-NOI domain of RIN4. The models were analysed with Swiss-PDB Viewer.

## Supporting information

S1 Fig Phylogenetic trees of short one-NOI RIN4-like/NOI family proteins from monocot species and PAS species. Bootstrap is shown in each node.
(TIF)

S1 Dataset IUPred score per amino acid position and predicted phosphorylated residues in all sequences analyzed. (XLSX)

S2 Dataset Amino acid sequences of all analyzed RIN4-like/NOI family proteins. Two-letter abbreviations for each species are included.
(PDF)
