# Supplementary figures and images for: Comparative and evolutionary analysis of Arabidopsis RIN4-like/NOI proteins induced by herbivory
Source: PLoS One. 2022 Sep 27;17(9):e0270791. doi: 10.1371/journal.pone.0270791 (PMC9514647; doi:10.1371/journal.pone.0270791)

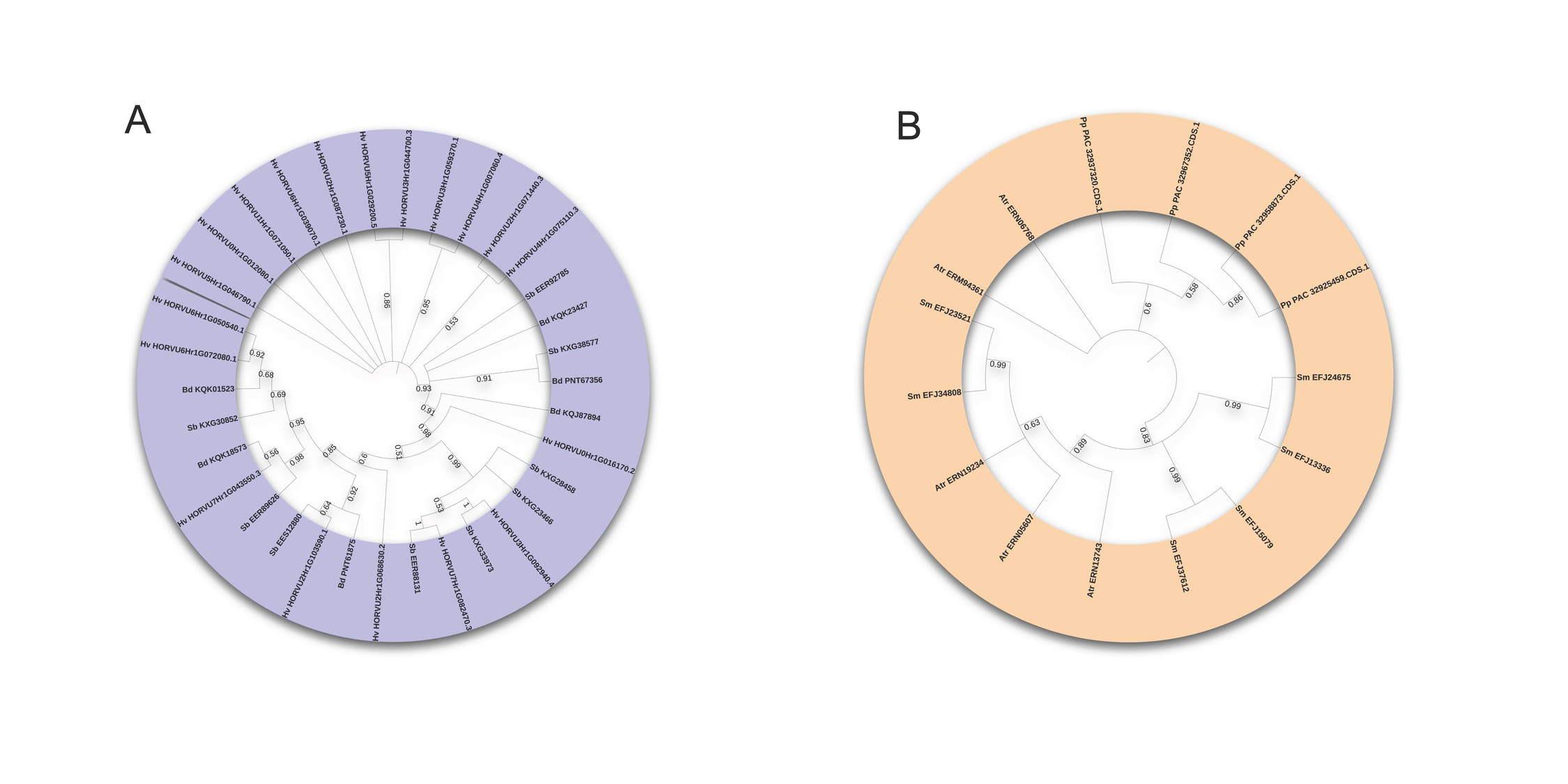

Supplement: S1 Fig — Bootstrap is shown in each node. (TIF) [file pone.0270791.s001.tif]
