# Supplementary material for: Comparative and evolutionary analysis of Arabidopsis RIN4-like/NOI proteins induced by herbivory
Source: PLoS One. 2022 Sep 27;17(9):e0270791. doi: 10.1371/journal.pone.0270791 (PMC9514647; doi:10.1371/journal.pone.0270791)
Supplement: S2 Dataset — Two-letter abbreviations for each species are included. (PDF) [file pone.0270791.s003.pdf]

Species used

Atr Amborella trichopoda
Ah Arabidopsis halleri
Al Arabidopsis lyrata
At Arabidopsis thaliana
Bd Brachypodium distachyon
Br Brassica rapa
Cc Coffea canephora
Gm Glycine max
Gr Gossypium raimondii
Hv Hordeum vulgare
Mt Medicago truncatula
Na Nicotiana attenuata
Pp Physcomitrium patens
Pt Populus trichocarpa
Sm Selaginella moellendorffii
Sl Solanum lycopersicum
St Solanum tuberosum
Sb Sorghum bicolor
Tc Theobroma cacao

Short one-NOI dicot

Group 1

>Al_fgenesh2_kg.8__1482__AT5G55850.1
MSDKGRPLPKFGEWDVNDPASAEGFTVIFNKARDEKKTGGKPGSPGKSSEGHAKSGGGDPSKPQP
VKKMALLHAISSCGLLTDTKMDLLAAQRMSQCLHILL

>Al_fgenesh2_kg.5__1197__AT3G48450.1
MATGNRARPLPKFGEWDATNPASAEGFTVIFNKARDDKKTKKTAVAGPESIVSPPRNEEPPKNNNN
HHNRNSQTPRSKKKWLCFR

>At_AT5G55850.1
MSDKGRPLPKFGEWDVNDPASAEGFTVIFNKARDEKKTGGKPGSPGKSSEGHVKSGGGDPSKPQP
VKKMALLHASSSCGLLTDTKMDLLAAKKKVTVLTHLKAMVLFMFYCRLS

>Ah_g09239.t1
MSDKGRPLPKFGEWDVNDPASAEGFTVIFNKARDEKKTGGKPGSPGKSSEGHAKSGGGDPSKPQP
KKWLCCMQSPAVDS

>Br_Bra002871.1-P
MEQDKGRPLPKFGEWDVNDPASAEGFTVIFNKARDEKKTGGKPGSPGKSTEGHAKSGGGGGGGDP
SKPQPKKWLCCMQSPAVDS

>Br_Bra035591.1-P
MSDKGRPLPKFGEWDVNDPASAEGFTVIFNKARDEKKTGGKPGSPGKSTDGHAKSGGGGDPSKPQ
PKKWLCCMQSPAVDS

>Br_Bra028957.1-P
MSDKGRPLPKFGEWDVNDPASAEGFTVIFNKARDEKKTGGKPGSPGKSTDGHAKSGGGGGDPSKP
QPKKWLCCMQSPAVDS

>Pt_PNT12596

MSDTGRPLPKFGEWDVNDPASAEGFTVIFNKARDEKKTGGQPESPGKVVDSHVKPGLNPA
KSQPKKWFCCIQSPHVES

>Pt_PNT58730
MYTDSCVQDTGRPLPKFGEWDVNDPASAEGFTVIFNKARDEKKTGGKPESPGKVDDSHVK
SGVNPAKPQPKKWFCCIQSPPADS

>Gr_KJB45528
MSDQGRPLPKFGEWDVNDPASADGFTVIFNKARDEKKTGGKPESLEKADTHGKPGADPNK
PQSVKKMALLRSSPAGGILKNSCRYKSFHCMMKPPPYILNKIGMNGGHLIKLFWDWI

>Gm_KRH46393
MSEKGRPLPKFGEWDVNDPASAEGFTVIFNKARDEKKTGGNPESPGKTATDPHSKPAVEP
GKTQTKKWFCCMQNPSAES

>Gm_KRH04752
MSEKGRPLPKFGEWDVNDPTSAEGFTVIFNKARDEKKTGGNPDSPGKIATDPHSKPAVEP
GKT

>Gm_KRG98467
MSEKGRPLPKFGEWDVNDPTSAEGFTVIFNKARDEKKTGGNPDSPGKTATDPHSKPAVEP
GKTQTKKWFCCMQNPSAES

>Gm_KRH33262
MSEKGRPLPKFGEWDVNDPTSAEGFTVIFNKARDEKKTGGNPDSPGKTATDPHSKPAVEP
GKTQTVRCS

>Gm_KRH04882
MSEKGRPLPKFGEWDVNDPTSAEGFTVIFNKARDEKKTGGNPDSPRKTATDPHSKPAVEP
GKTQTVRCS

>Gm_KRH04877
MKEKGRPLPKFGEWDVNDPTSAEEFTVIFNKARDEKKTGGNPDSPGKTATDPHSKPAVEP
SKTQTVRCS

>Na_OIT30867
MSEKGRPLPKFGEWDVNDPASAEGFTVIFNKARDEKKTGGKPESPSKADGNTKQGEEPLK
PQTKKWFCCMQSPHAES

>Sl_Solyc07g065640.3.1
MSEKGRPLPKFGEWDVNDPASAEGFTVIFNKARDEKKTGGKPDSPSKTDGNRKEGEEPLK
PQTKKWFCCMQSPHAES

>Cc_CDP13707
MSEKGRPLPKFGEWDVNDPASAEGFTVIFNKARDEKKTGGKPESPSKADAAAKQGVDPVK
PQGKNWFCCMQSPHAES

>Gm_KRH14215
MSDTGRPLPKFGEWDVNDPASAEGYTVIFNKARNEKKTGGKPDSPAKVNNPRTRPPLDPS
KTQSKKWFCCIQSPPAES

>Gm_KRH73917
MSDTGRPLPKFGEWDVNDPASAEGYTVIFNKARNDKKTGGGKPESPAKVNPRTRRPPLDP
SKTQSKKCFCCIQSPPVES

>Mt_AET00799
MDKGRPLPKFGEWDVNDPASAEGYTVIFNKARDEKKTGGKPESPAKANTQTRPPLEHAKT

HGKSWFCCLHSPPAES

>Tc_Tc07v2_p001030.1
MSDKGRPLPKFGEWDVNDPASAEGFTVIFNKARDEKKTGGKPESPAKSDTHVKSGVDPGK
PQPKKWFCCIQAPAAES

>Gr_KJB81610
MSEKGRPLPKFGEWDVNDPASAEGFTVIFNKARDEKKTGGKPESPSGGDPHVKPGADPSK
TQPKKWLCCMHAPPAES

>Gr_KJB13906
MSEEKGRPLPKFGEWDVNDPASAEGFTVIFNKARDEKKTGGKPESPAKSDSNVKPGADPS
KTQPVKKMVLLHSSPACGILTMLYSYKT

>Gr_KJB63074
MSEDKGRPLPKFGEWDVNDPASAEGFTVIFNKARDEKKTGGKPESPAKADAHGKPGADPS
KPQSKKWLCCVQAPPAES

>St_PGSC0003DMT400028291
MQVRGFELGLPAHVVAPLPYVARTLQKCQWEKGHPLPKFGEWDVNDPASAEGYTVIFNKA
RNEKRSGGNTNSPPKGDPTYKHGATLGKPQSNRDLSLLLPAALESV

>Sl_Solyc07g045340.3.1
MAEKGQPLPKFGEWDVNDPSSAEGFTVIFNKARNEKKTGGKVDSPPKGDSAYKNKATLGK
PQSKKWFCCMQSTAAES

>St_PGSC0003DMT400044780
MAEKGQPLPKFGEWDVNDPSSAEGFTVIFNKARNEKKTGGKVDSPPKGDSAYKNKSMLGK
PQSVSRGPHNGC

>Cc_CDP20461
MPRCGPHFTASCFDSSSPSPVLSSSSFIFISILISCGEGEKKGLKEREREISNSYQSYRH
SYSTPIFLPPQLPIMAEHDRPLPKFGEWDVNDPASAEGFTVIFNKARNEKKTGGKSDSPQ
KGSSAYKHRATLGKPPSKKWFCCVQSVATE

>Pt_PNT05357
MIKISVTNTIKSMYVCLSMWPCCFFLNQVVFQNEPCDKEKDQPLPKFGEWDVNDPASAEG
FTVIFNKARNEKKTGGKPDSPAKDSSTYKPGATTTLGKPQTKKWFCCIQATHAE

>Tc_Tc01v2_p016460.1
MTNQKLIRIIRKEKGGEAQTPERFSASLPNEIMADKGRPLPKFGEWDVNDPASAEGFTVI
FNKARNEKKTGGKVDSPGRKDPTYKQGAVLGKPQSQKKWFCCIQAAHAD

>Gr_KJB42728
MADKGRPLPKFGEWDVNDPSSAEGFTVIFNKARNEKKTGGKIESPGANDPACKQEAVAAK
PQVKKMVMLRTSYSCRLKRWLLRC

>Br_Bra013208.1-P
MVVISEITDDDNGDKKPQEPPGGKTRIITTTWPMVSNVRIDFVTYAVVKQRIYTLSPPSTCVVSRLRR
RQGGEIRGKVESVEVVFKTTKSTPSIELSRTVIDKGRPLPKFGEWDVNDPSSAEGFTVIFNKARNEKK
GGGVSDSPRKDEPPGFNKNGYVFGKPTVSDSISSLNSRKVKIVER

>Al_fgenesh1_pm.C_scaffold_3002274
MADKGRPLPKFGEWDVNDPSSAEGFTVIFNKARNEKKGGGKSDSPGKDEPGYNKNGEVLEKPAKK
WFCCI
RAE

>Ah_g16454.t1
MADKGRPLPKFGEWDVNDPSSAEGFTVIFNKARNEKKGGGKSDSPGKDEPGYNKNGEVLEKPAKK
WFCCIRSE

>At_AT2G04410.1
MADKGRPLPKFGEWDVNDPSSAEGFTVIFNKARNEKKGGGKSDSPGKDEPGYNKNGEVLEKPAKK
WFCCIRAE

>Na_OIT01710
MAEGRPLPKFGEWDVNDPASAERFSVIFNRARNEKKTGKIDHSPPKKNYKHTATLGKPQS
KKWFCCMRSSDAD

>Sl_Solyc12g006080.2.1
EAGRPLPKFGEWDVNDPATAEGFTVIFNKARNEKRSGGKADAPPKSNHKHTATLGKPQSK
KWFCCMRSGAVE

>St_PGSC0003DMT400010963
MEEAGRPLPKFGEWDVNDPATAEGFTVIFNKARNEKRLGGKADAPPKSNHKHTATLGKPQ
SKKWFCCMRSGAVE

>Gm_KRH74047
MAERGRALPKFGDWDVNNPSAAQDFSVIFNKARNERKTGANKIHFPPNHNNTTKCNPPQV
VLGKSHYKKWFCCINTSAES

>Gr_KJB38745
MAEKGRALPKFGEWDVNNPAAAEGFTVIFNKARDEKKTKGSAPNIMPSASSKKNQPPPSP
PTPKGKWFCCVGV

>Gr_KJB51162
MAEKGKALPKFGEWDLKDPATADGFTVIFNKARNEKKTRATAPPPPSIVPQKVDTVNKPP
PSPPKSLKGSI

>Pt_PNT01204
ITNKHNVCSFSIFSPFPLFLLPKISLFLAPFSSIKLQPSQRSFPSFNHSSVLFCSVLFFL
MASQGRPLPKFGEWDVNNPASAEGFTVIFNKARDEKKTKNSPEKVVSPRRTEPGYNKNDK
NENYKHPPKRRWLCCS

>Pt_PNT10292
QGRPLPKFGEWDVNNPASAEGFTVIFNKARDEKKTKNSPAKVVSPRRTEPVFNKNAKNEN
YEHPPKRRWLCYVELIMH

>Gm_KRH03903
MSSENGRPLPKFGEWDVNNPASAEGFTVIFNKARDEKKTNTATATPTPRRSDPVFKNENY
NNPQYSGKRKWFCCG

>Gm_KRH57184
MASENGRPLPKFGEWDVNNPASAEGFTVIFNKARDEKKTNTATPRRSDPVFKNENYNTPQ
YSGKRKWFCCG

>Ah_g22168.t1
MASNEAGRALPKFGEWDVNDPATADGFTVIFSKAGEDKKTGRSSTKTNSQRKQNGDKPAVKKWL
CFTFA

>Al_scaffold_304083.1
MASNEAGRALPKFGEWDVNDPATADGFTVIFSKAGEDKKTGRSSTKTNSQRKQDGDKPAVKKWL
CFTFT

>At_AT2G17660.1
MASNEAGRALPKFGEWDVNDPATADGFTVIFSKAGEDKKTGRSSTKTNSQRKQDGDKPAVKKWL
CFTFA

>Br_Bra002076.1-P
MASNDAGRPLPKFGEWDVNDPATADGYTVIFSKAGEDKKTGRSSTKTASQRKQDGDKPVVKKWL
CFTFS

>Br_Bra024499.1-P
MAANDAGRPLPKFGEWDVNDPATADGYTVIFSKAGEDKKTGRSSTKTNSQRKQDGDKPAVKKWL
CFTFS

>Br_Bra037263.1-P
MASNDAGRPLPKFGDWDVNDPATADGYTVIFSKAGEDKKTGRSSTKTNSQRKQDGDKPAVKKWL
CFTFS

>Ah_g00348.t1
MASNSEARPLPKFGEWDVNDPATAEGFTVIFSKAGEDKKTGRSSSKAPSQRKQDGVKPTAKKWLC
FTFS

>Al_scaffold_700550.1
MASNSEARPLPKFGEWDVNDPATAEVFTVIFSKAGEDKKTGRSSSKAPSQRKQDGVKPTKKWLCF
TFS

>At_AT4G35655.1
MASNSDARPLPKFGEWDVNDPATAEGFTVIFSKAGEDKKTGRSSSKAPSQRKQDGVKPTKKWLCF
TFS

>Ah_g16697.t1
MATGNRARPLPKFGEWDATNPASAEGFTVIFNKARDDKKTKKTAVAGPESIVSPPRNEEPPKNNNN
HNRNSQTPRSKKKWLCFR

>Gr_KJB17526
MAASEERERTLPKFGEWDVNDPATSEGFTVIFAKASDEKKAAKMTGSIPSKKRSQNSNST
KQPEKKNWFCCMSA

>Tc_Tc01v2_p000690.1
MASDDRERPLPRFGEWDVNDPATAEGFTVIFAKASNDKKAAKASGNTPSKKRYKDSDKQS
DKRKWLCCISA

>Mt_AET04509
MASYENNGRPLPKFGEWDVNDPASAEGFTVIFNKARDEKKTGGGGSGRVNSQRLRSNSRK
DDDKSSKKKWFCFKP

>Gm_KRH31389
MASYDEKQGKPLPKFGEWDVNDPASAEGFTVIFNKARDEKKIASASGRFPSQRRYDSRKH
KDQQKCKSSSSTSKKKWFCFGP

>Gm_KRG97496
MASFDEQQGKPLPKFGEWDVNDPASAEGFTVIFNKARDDKKIASASGRFPSQRRYDSRKH
GKDKKKCKSSSSSSSSKKKWSCFGP

>Mt_AES72107
MYVQYSEQDGKPLPKFGEWDVNDPSSAEGFTVIFNKARDDKKIASASGRFPSQRRYDSRT
GRTNDNNKNLKASSKVPNNKLVLLMTIINNKLC

>Br_Bra020256.1-P
MRNGCLAKLRSKYLGKMCNKGEITSEKSKEEWKPKVEECWKRKEVKDKSQVHDPSTANTNISSRE
NQVIKEMADKKFDDTPALYKSMDTEKDENSNEKQTLERGSGKVDVNRTKAGQDSTQDQPKPSRVL
PKFGEWDVRDPVTADVFRVIFNKPKDERRGALNDEFYKVKTFDLRGIKSGGRK

>Sl_Solyc02g093670.3.1
MNSQEKGRPLPKFGEWDVNNPASADGFTVIFAKARDDKKANSSAAPTQTPRNDYAYGQPN
PYQQETRKRRFCCF

>St_PGSC0003DMT400032859
MEQEKGRPLPKFGEWDVNNPASADGFTVIFAKARDDKKANSSAAPTQTPRNDYAYGQPNP
YQQETRDVSVAVNYESLRPSGIAAQVCQVGE

>Na_OIT05937
QEKGRPLPKFGEWDVNNPASADGFTVIFAKARDDKKANGTAAPTQAPRNDYAYGQSNPYQ
QEPKKKWFCCF

>Na_OIS98792
MTSQEKGRPLPKFGEWDVNDPASADGYTVIFAKARDEKKATGTSASSVPAASPRAEAESR
QNDSCQQDPTKKWFCCF

>Al_scaffold_0007_3599
MASNNQQRQQQDRPLPKFGEWDVNDPASAEGFTVIFAKARDDKKTNASGRATSQRRDNNNKSQD
EPTKKRFCCF

>At_AT5G40645.1
MASNNQQRQQQDRPLPKFGEWDVNDPASAEGFTVIFAKARDDKKTNASGRAASQRRDNNKSQDE
PTKKRFCCF

>Pt_PNT58098
MSSQDQGRPLPKFGEWDVNNPASAEGFTVIFSKARDEKKSGAAAGAGAASQRKTNSSQAN
SQCPPPKKRFCCF

>Br_Bra029233.1-P
MATENKGRPLPKFGEWDVNNPASAEGFTVIFSKASDEKKTKKASGAVPNSQRNQNSDQNNHHDSQ
NSKPKIANLILCSLSRRNGFASVDSCLLLKNPRIMKLNKVNPQEATHLSRLDPIHLQTEAPINVERED
QTCIFKLFPRVPVPPHLVDVSDDEDV

>At_AT5G63270.1
MAENKGKPLPKFGEWDVNNPASAEGFTVIFSKASDEKKTKKASGAGPNSLVSPQRNQNSDQNNNH
SSQNPKAKNKWFCFR

>Ah_g12334.t1
MAGNKGKPLPKFGEWDVNNPASAEGFTVIFSKASDEKKTKKASGAGPNSLVSPQRNQNSDQNNHH
DSQNPKAKNKWFCFR

>Al_scaffold_0008_2859
MAGNKGKPLPKFGEWDVNNPASAEGFTVIFSKASDEKKTKKASGAGPNSLVSPQGNQNSDQNNHH
DSQNPKAKNKWFCFR

>Br_Bra018058.1-P
MATGNKGRQLPKFGEWDATNPASAQGFTVIFNQARDDKKTKKTAVAGPESLVTPPVNIEPQRRHN
HHNNKRNPQIQREKVPSSFHPQNQILTKKESRLLTDISFFSVLQKGWLCFR

>Br_Bra019544.1-P

MATGNKGRQLPKFGEWDATNPGAAQGFTVIFNNARDDKKTKKTAVAAPESLVTPPTNNEPHQNN
NHRHHRHQQNNNHRKKRNSPPPREKVSSFFPPNPNLDLRVDYSRDIMFAEEMALLPLSPVRALFPK
RNDKEADRLSRVVFNK

>At_AT3G48450.1
MATGNRARPLPKFGEWDATNPGSAEGFTVIFNKARDDKKTMKTAVAGPESIVSPPRNEEPPKNNNN
HHHNHHTRHSQTPRSKKKWLCFR

>Gr_KJB31051
MSSYGKGQALPKFGEWDVNDPASAEGYTAIFNLARDEKRTGGNVTVVATEGIKSQQKQGA
RKEKRKHKYCIKVKTHKFKDYLGSHFYKNSPH

>Mt_KEH31733
MRPYLWGYKHLIECLKEQDKGRPLPKFGEWDVNNPASAEGFTVIFNKARDEKKTNTSINM
ATPRTADPVFKNDNHPKYPRKGLDNKSNLQTLNCYVEMFLLDHKLIYC

>Mt_AET00994
MSSPKFGEWDANDSSSANGYTVIFNKIKEEKRGGKFQSPPNNSASNAHKRKDFLGKTLSV
SSFFFFPLYLLLSLYNLIFYFLKDFFFCHSATICWHVG

>Mt_AES64417
MENPEIFNKHVNVDEVKPSHTYSHKASSTEKGSHEVPKNNSTHHLHRRRSRGSKGSFTSE
FGSEKSHIDHSVINKTSQSEHKRSVSKGIGSNTGSFSSSNHRSESRSFNDHGDHRAVAIP
EFGKWDVTDPKSGEGYTVMFSKIKEEKQIMSSRISGLRTTPHNNGSNIKNQHDGSSFNLS
KYCCCLSTSESK

>Gr_KJB68566
MNKHDETATIPPFGEWDETDPTSGQKFTVIFDKKKEEKNDPSFTFPISAPPKSSRSSNFL
RSKRPSPSFYSKICCCLSAR

>St_PGSC0003DMT400086804
MINPNDPEENPEAFACCGDEDASINISPTKTPLVEKHQYHYGHRRNPSAESGQNKSIGPT
NSNSESFGDSQSISGFSVNQPTRRRRTSDVKKNKNDRGNGFVPPSPNRLMKNSRNPSDDL
SCSSAASVPKFGAWDEKDPKSGEGFTVIFNKVKEEKHIAAAKFPIVQPQSNMSSSNNRKK
NAKSKVFCCLF

Group 2

>Pt_PNT56938
MERRRKNGGLSVPQFGGWNSKNPVPTDYSVVFTQARANRRQHKSDVRHASLGNERELLAA
ANQQEDSVMKRKKFLTYINCCIRP

>Pt_PNT20004
MYLRTDNLYSQSYFHSLIMAMGEKNGGLSVPQFGAWDSKNPVPTNYSMVFTRARANRKQH
KSDVRHASLGNERELLAAACQQEEPVMVSLQSTLYLLCTV

>Gr_KJB35708
CSPPSPILLLVTMAHQTENNVVGWTPVPQFGAWDQKNAGATDYSMIFNQARANRKQHKSD
VRRSLGNDRDLVVSSFPKRPPDDHYPVSKKKKILTYINCCIKP

>Gr_KJB21812
MTTNKNENKKWMSVPEFGGWDKQPPGATNYSMVFSQARANRKQQKSDVFRNLGNHRDFTA
DSSPQPPQPHQQADNNDQDSAVTNYAWMLVPEFRGCDRKSTGSMDYSTVFSKARANRKRQ
MSGDWRSLGNDDDFITVSLPHEQPPYQKKHKVVCLRKLNCTFFFLSRIRITKLISITMQK
RNKIFAFLLCCFWQ

>Tc_Tc09v2_p020540.1
MAHRTENNAWMSVPQFGGWDQKGPGATDYSMVFSRARANRKQQKTDIRRSLGNEQEFVAA
ASLPQPQYQEDSVTKKNKILTYINCCIRP

>Br_Bra006506.1-P
MQNAGWTPVPQFGGWDQKGIDATDYSVVFTKARANRKLNKADVSHSLGSEQELMASARRH
HYQQHLNNRRETQDDDPVMKKKRILTYINCCIRPN

>Ah_g13119.t1
MFGSLSKLYICLSDEFYSWLQNAGWTPVPQFGGWDQKGPNDATNYSVVFSQARANRKQNKAGVR
HSSLGSEQELMASIRRNHQQLHHRHETQDDDPVMSGSLALFWHDDWTGLGPLIDLIGLNGPMVTGI
STTSSVSLVIANGSWLLPRGRHPLLLLLRACLPATPPTLSPTISDYFLWRNSHSDTPGLFSSSKTWASL
NSQSLVQVSWFKSVWFSQRIPKHAFILWVILRDRLTTRDRLGSWGLNVPGHCLLCSSSLESKQLLFF
DCVFSKEVWTAFLSHQNLSPPTLFDDIVHWVRSPTSNARLNIICKLIFHAVVYFIWIERNSRIHTNIAR
SAIILVKEIQLTLRAKLAGLDRRKVLLHSSLAAPSRQPQESYLSTWFRFIQL

>Al_fgenesh2_kg.6__1972__AT5G19473.1
MDKRRENAGWTPVPQFGGWDQKGPNDATNYSVVFSQARANRKQNKAVVRHSSLGSEQELMASV
RRNHQQLHHRHETQDDDPVMKKKRILTYINCCIRPN

>At_AT5G19473.1
MDKRRENAAWTPVPQFGGWDQKGPNDATNYSVVFSKARANRKQNKAGVRHSSLGSEQELMVSV
RRNHQQLHHRHETQDDDPVMKKKRILTYINCCIRPN

>Mt_AET00188
MDQKQGMKKHAKSSVPQFGAWDQKTMGATNYSMVFTQARANKKQQKTDLTEVKRSSIGTE
EELVKAINHRHGHPPQGYHAQPAHGRAPPAQVQANAQVHANANANAQVQEDPVVMGKRRI
LTYINCCIRP

>Gm_KRH10571
MEQKQQRPAMMSVPQFGGWDQKAPGATDYSMVFTQARANKKQQKTNLTEIKHRSLGNERD
IANAKHGKAHHHHHVHGHSHAHEEPLVVGKKKILTYVNCCIRP

>Na_OIS96482
SKPFQPFLSDLFLFVLHMEKRKEKNGPTYVPQFGAWDNKAAAGGNSSYTIEFSKARANKQ
QQHKNGLGARHGLANDQQPQHKNGLGVRHGLTNDQEFPGKKQEDPSNMKNGYRSVPQFGM
WDQQAGGAASYTVEFSKARVNRKQHKNNDLLARPPSIDHEQEALRKQHEDSCMRKKKMLA
FCCIGP

>Na_OIT18857
PKKIPPFSLFFFLSNFSSNMAKQREKYGLPYVPQFGAWDHKTGDNLNFSMVFSQARANKK
QNRHNLAQHNLGNEQEILAKLQEVSPREDSSTPVPQFGERNQKTEGNPDYSKVSPKAHAN
KKSHRHDLTHRRLGSREETR

>Gm_KRG50626
MERKGKKPVKMSVPQFGGWEHKPAGVPTDYSMVFNQAREKKKTQMNDLTEVKRLSLGNER
DALKPNHRRGHGQGHGHGHGHGHGHGHGHGHGLDHEDPPVMRRKMVLNYMNCFKP

>Mt_KEH33367
MSILCIRKHLTSDPCYGPLYYEMPVLIDNLLPASVVSEPQLKAGVHMQKPKMSVPQFGGW
ENKSKGVPTDYSMVFNIARENKKNHKTDWSEVKRRLSISNERVATNATNHRHGHGHGHGF
GHNHHHHSHADSPVMGRSNALSYMNCCLRP

>Ah_g19170.t1
MEDDRKEKNTPWLSVPQFGDWDQKGGGTMPDYSMDFTKIREMRKQNKRDPSRASLGNEEELIKPP
ESATSTANLTTVQSENQREFSPSHHHHQPHSPSTRRSIFSCFNCCVKA

>At_AT5G09960.1
MEDDRKEKNTPWLSVPQFGDWDQKGGGTMPDYSMDFTKIREMRKQNKRDPSRASLGNEEELIKPP
ESATSTAELTTVQSENQREFSPSHHHQPHSPSTRRSMFSCFNCCVKA

>Al_scaffold_601004.1
MEGDRKEKNTPWLSVPQFGDWDQKGGGGTMPDYSMDFTKIREMRKQNKRDPSRASLGNEEELIK
PPESATSTANLTTVQSENQREFSPSHHHHQPHSPSTRRSIFSCFNCCVKA

>Br_Bra028607.1-P
MEEDRKEKNSPWLSVPQFGDWDQKSGGGAMPDYSMDFTKIREMRKQNKRDPSLASFGNEE
ELIKPPESSAPIAKLTTVHTENKQHFSPDHHHQPHSPSAKRSIFSCFNCCVKA

>Br_Bra009071.1-P
MYQETQQLYQLWRLAIQERDEAREQLMLLLAELSQLRELLNTVLLSKEKIGSYYLEAADESTGHQN
CSYNLFPGDSPSNFSVNSCPLDLSVLSNQMRFVVENMRDYETVVLEMIGGVLPENGFNQMEDDRKE
KNSPWLSVPQFGDWDQKGGGTIPDYSMDFTKIREMRKQNKRDPSRASLGNEDELVKPPESATTATA
KLTTVHSENQHHFSPAHHHQPHSPSTRRSIFSCFNCCVKA

>At_AT5G64850.1
MEDRKENKNSPWLSVPQFGDWDQKGGSIDYSMDFSKIREMRKLNKRDASRASLGNEEELINPFHD
QPPVDTAKPKKLTTVHSDNNNRNEFSHHHPHSPSRRRRIFSCFNCCVKA

>Al_fgenesh2_kg.8__2521__AT5G64850.1
MEDRKENKNSPWLSVPQFGDWDQKGGSIDYSMDFSKIREMRKLNKRDASRASLGNEEELINPFHN
QPPVDTTKPKKLTTVHSDNNNHNEFSHHHPHSPSRRRGIFSCFSCCVKA

>Ah_g07486.t1
MEDRKENKNSPWLSVPQFGDWDQKGGSIDYSMDFSKIREMRKLNKRDASRASLGNEEELINPFHN
QPPVDTTKPKKLTTVHSDNNNHNEFSHHHPHSPSRRRGIFSCFNCCVKA

>Br_Bra037798.1-P
MEDPKENKNSPWLSVPQFGDWDQKGGSIPDYSMDFSKIREMRKQNKRDPSRASLGNEEEL
INPFHNQPASVDNTKPKLTTVHSDNNKTHNEFSHHHPHPPSVRLWNLTLRGEESSAASTA
ALKLEEMKNGNVATYYCGSRH

>Br_Bra024332.1-P
MDDLKENKNSPWLSVPQFGDWDQKGGTIPDYSMDFTKILLEPVLATKKTSLTLFITNLLQ
LIILSLNSRLFTATTTEPTLSSLTTSHFLHL

>Br_Bra031897.1-P
MEDQKENKNSPWLSVPQFGDWDQKGGPVPDYSMDFSKIREMRKQHKRDPSRASLGNEQDF
INPFHNQPTSVDKTKTKLTTVHSDNNITNNEFPHHQRHPPSVLTEFLSMKWMIVFVDSSY
VYSFDVGLDFFGWLDYEMASSSNLVTVVLQCLV

>Gm_KRH15631
MVPKPSTLQPYSLTHSHGTSTPRSHSRSCSWCQSCSCSHSHLSTAEKIHAATIVPIYHHF
ISLANLSNFLSRSEITLMEQSKEKNTTWLLVPQFRDWDQKGQVPDYSFDFSKIREMRKQN
NTNISRASLGNEEKLMASSTTSNTSTIHSDDHQHPYYHQTNSPTIRSLSFLFY

>Gm_KRH64143
MEQSKEKNAPWLSVPQFGDWDQKGQVPDYSLDFSKIRETRKQNKTNISRASLGNEEELTA
SATNNANTVHSDEHQHPHYNQTNSPTVRSLTCFIILILLV

>Gm_KRH43319
MDRRKEKNAPWLSVPQFGDWDQKGQVPDYSLDFSKIRETRKQNKTNISRASLGNEEEFMD

STTSSSVNTAHSSEHPQPHYHQTHSPTVRFLSTQAA

>Gm_KRH59453
MDRRKEKNGPWLSVPQFGDWDQKGQVPDYSLDFSKIRETRKQNKTNISRASLGNEEEFMD
STTSSSVNTGHSSEHPQPHYHQTNSPTVRFLSSQASLFFIFGKLFNTITVDPHKYCSYSL
YVASKSSSV

>Mt_AET04587
MPMDARKEKNGGWLSVPQFGDWDQKGQVPDYSLDFSKIRETRKQNKTNISRASLGNDEEF
NIDSTSSSTSNTVSSIEQQQQQHTPRYHHQTHSPTTRKSFLSYFNCCVKS

>Gm_KRH53801
MEQSKEKNAPWLSVPQFGDWDQKGQVPDYSLDFSKIREMRKQNKTNISRASLGNEEELMA
SSATNNTSTVHSDDHQHPHYHQTNSPTTRRSFLSYFNCCIKA

>Gr_KJB18339
MEDRKEKNAPWLSVPQFGDWDQKGQVPDYSLDFSKIREMRKQNKREVSRASLGNEEEFIN
PTANTVSTTPSDDHHHNYPQNHHSPSTRRSFFSYFNCCVKA

>Gr_KJB49118
MEDRKEKIGAWLSVPQFGDWEQKGQGQLPDYSLDFSKIREMRKQNKREVSRASLGNEEEF
INPTATTVTTAPTTDDHHHYPPTHHSPTSKRGIFSYFNCCIKA

>Tc_Tc01v2_p009760.1
MEDRKEKNAPWLSVPQFGDWEQKGQLPDYSLDFSKIREMRKQNKREVSRASLGNEEEFIN
PTATSVSTAPSDDHHHYPQNHHSPTTRRSILSYFNCCVKA

>Pt_PNT27737
MEDRKDQKNAPWLSVPQFGDWDQKGELPDYSLDFSKIREMRKQNKKDVSRASLGNEEELI
NPTATAAKPAQTQDHHRHHHYHEGHHHSPTIGTFLDKNLDHKKREELERNHTRRSIFSYF
NCCVKARG

>Gm_KRH73533
MERQEIRPPVMSVPQFGGWDQNEPGAIDYTVMFTQARENKKHQKANLIEIRRKSFGSERD
FVNSNHGRAPRHHVRSHTHSHAHEDHVVMGKKRILTYINCCIRPTIAAT

>Pt_PNT35630
MEDRKDQKNAPWLSVPQFGDWDQKGELPDYSVDFSKIREMRKQNKRDASRASLGKEEELI
NPTATTAKTAQTHDHRHHYHQDHHDSPATRRSIFSYFNCCVKA

>Mt_AES65151
MERHSEENSPCPPVPRFGEWDQKGPIRDYSMDFSKIQEARKQLKSLGNEEELKASFRHIQ
RQRTEHDASPTRRKSFLKWFSCCIKF

>Mt_AES65148
MERHTEENSPCPPVPRFGEWDQKGPMRDYSMDFSKIQEARKQHKSLGNAEELKASFQHIQ
RQRSEREASPMRRKSFMKWFSCCIKF

>Mt_AES65146
MERHTKENSPCPPVPRFGEWDQKSPIRDYSMDFSKIQEARKLEKSLGNEEELKASFRHIQ
RQRSERDSSPTRRKSFMKWFSCCIKF

>Mt_AES72738
MQKNAPYQSLPQFEKWIQKPPFSDSSMYNSKIKKTKKPNRGASLGNEEEFKVHIMKNKKT
EETNISKKRRNLLKYFSCCIKF

>Mt_AES72742

MEQNKEKIAPWLSVPQFGEWDQKGPLPDYSMDFSKIREMRKQNKTNASRASLGNEEEFAA
PTQKNVKADHSEPQHPHYHKTNSAVRRRSFMSYFNCCIKA

>Sl_Solyc05g051710.3.1
MEDHKERAAPWLSVPQFGDWDQKGALPDYSVDFSKIRENRKQNKSRASLGNEEDFNSISN
TSNNNVNNTSHSTQNNDQTYHHKHSTTHTRKSIFSCFNCCVKA

>St_PGSC0003DMT400077395
MEDRKEQKAAPWLSVPQFGDWDQKGALPDYSMDFSKIRENRKQNKSRASLGNEEDFNSIS
TTSNNNNVNTSHSTQNNDQTYHQKHSPTHVRFCDINSFIYPCFLLS

>Na_OIS98501
MEDSKEKTAPWLSVPQFGDWDQKGALPDYSLDFSKIRENRKQNKRDLSRVSLGNEEELVS
STTSNANTGSSAPSNDQNYNQNHSPTVRFCDINSLILFLLRLFGPIFSLKC

>Na_OIT19386
QKTGAWLSVPQFGNWDQKGAFPDYSMDFSKIRENRKQNKKDLSRASLGNEKELIFTTKKD
VNSGYSPHSNDHHYHQNRCPTRMRKIFNYFNCC

>Sl_Solyc04g011910.3.1
MEYHKEKKNGPWLSVPQFGHWDQKGVYPDYSMDFSKIRENRKQNKKDLSRASFGNENDLI
FSTKSDANENDHHYHQNQSTTRMRRIFRYFNCCGKA

>Na_OIT27281
RYNTYPQHTAKSPCIFAKFPYFPASSSYQIACFLISKTPFFFLSQFTLQTPVCIFVHPSV
SAMDDRKEKTAPWLSVPQFGDWDQKGVMPDYSMDFSKIRENRKQNKRDLSRASLGNEEEL
ISSTNKNANTGHHHSAHSDDLHFHQNHSPTTRRSIFSYFNCCVKA

>Cc_CDP12895
MDYRKEKNTPWLSVPQFGDWDQKGVMPDYSMDFSKIRENRKQNKRDPSRASLGNEEELVS
SATSNSIASHSDDHHHFHQNNSPTGRRSIFSYFNCCVRA

>Sl_Solyc04g051820.3.1
MDDRKEKTAPWLSVPQFGDWDQKGVMPDYSMDFSKIRENRKQNKSRASLGNEEELISTAN
SKSNTVHSARSDDLQFHQTHPSTTRRSIFSYFNCCVKA

>St_PGSC0003DMT400042669
MDDRKEKTAPWLSVPQFGDWDQKGVMPDYSMDFSKIRENRKQNKSRASLGNEEELISTAN
SKSNTVHSAHSDDLQFHQTHPSTTRRSIFSYFNCCVKA

Group 3

>At_AT5G18310.1
MAHGRYDTYKKKSGQIPRFGEWEEANEMPITQYFENPRQAEALKLASHHPRPRHLHAQRQTAGTK
EKRGPQRRVRDVSAQSDKYYIDVNGVKQFKNDVALTCKPPKPVDEDLYKIPPEFIHSSTRKRRPSFL
ACLVPCA

>At_AT5G48500.1
MDYEYGGGEYCRRGHVPAFGSWDWNDAVPFTQCFETATTQQPAFLHYASYPQDRDLYLAGDLYD
NHHLVAPAVIIVPRRRAKVGQEPAEKKRNVSKQQHNYKTEAREFNAPVSCPTPVVKRRMKAPKPV
DEDLYKVSPQLLSVKSKRKRGGGGFGCISRCFLPTRVL

>Ah_g15931.t1

MDYEYGGGEYCRRGHVPAFGSWDWNDAVPFTQCFETATTQQPAFLHYAPYPQDRDLYLAGDLYD
NHHLVAPAVIIVPRRRAKVGQEPVEKKRNVSKEQHNYKTEAREFNAPVSCPTPVVKRRMKAPKPV
DEDLYKVSPQLLSVKSKRKRGGGGFGCISRCFLPTRVL

>Ah_g13265.t1
MAHGRYDNYKKKSGQIPRFGEWEEANEMPITQYFENPRQAGLIRHHYTTTSSASSTTTTSSSSSSSA
EALKLASHRPRPRQTAGTKEKRGPQRRVRDVSAQSDKYYIDVNGVKQFKNDVAPTSKPPKPVDED
LYKIPPEFIHSSTRKRRPSFLACLVPCA

>Al_fgenesh2_kg.6__1838__AT5G18310.2
MAHGRYDNYKKKSGQIPRFGEWEEANEMPITQYFENPRQAGLIRHHYTTTSSASSTTTTSSSSSSSA
EALKLASHRPRPRQTAGTKEKRGPQRRVRDVSAQSDKYYIDVNGVKQFKNDVAPTSKPPKPVDED
LYKIPPEFIHSSTRKRRPSFLACLVPCA

>Al_fgenesh2_kg.8__727__AT5G48500.1
MDYEYGGGEYCRRGHVPAFGSWDWNDAVPFTQCFETATTQQPAFLHYAPYPQDRDLYLAGDLYD
NHHLVAPAVIIVPRRRAKVGQEPVEKKRNVSKENHSYKTEAREFNAPVSCPTPVVKRSMKAPKPVD
EDLYKVSPQLLSVKSKRKRGGGGFGCISRCFLPTRVL

>Br_Bra002152.1-P
MGTQGRYDNNYRKKSGQIPRFGDWEDANEMPITQYFENARQAGLLRHHHNFTTSSSSTAT
SSSSSSSAEALKLASHHPRPRHLHHSRQTAGTKEKRGPQRRVRDVSSQTDKYYVDVTGVK
HDVAPASRPPKPVDEDLYKIPPELIYSSRRKRRSGFLACLVPCAS

>Br_Bra036167.1-P
MDYEYRGEYCRRGDVPAFGSWNWNDAVPFTQCFETATTPQPAILHYAPYPQDRDLYLEDD
LYDSHHHHHLVSPAVILLPRRKSRVGQEPKRDTSKEEHNFKNEATRCPTPVEKRRMAAPK
PVDEDLYKVSPQVISSKSTKKRGGVFGCISRCFLPTSVRD

>Br_Bra020695.1-P
MDYEYGGGEYCRRGHVPAFGGWDWNDAVPFTQCFETATTQQPSYLHHYPPYPQDRDLYLA
GDLYDNHHLVAPAVILLPRRRAKVGQEPKRTSSKEQHKFKKDARESNAPRSCPTPVVKPR
TARPKPVDEDLYKVSPRLLSLKSTKKRGGGFGCISRCFLPTRVL

>Br_Bra037483.1-P
MDYEYGGGEYCRRGHVPAFGTWDWNDAVPFTQCFETATTTQQQPTFLQYAPYPQDRDLYL
AGGDLYDNHHLVAPAVILVPRRRAKVGQEPKRTSSKEQHNFKTEARECDAPTSCPTPVVK
RRTKPVDEDLYRISPTLLSVKSTKKRGGGFGCISRCFLPTRVL

>Cc_CDP04250
MEYFEGKRSGQIPAFGNWDSANELPITQYFENARQAGLVRCNVSALTTDNQHQYCNPYMT
SARASGGGGYPIDLCAADSQKPSVRLVAVPPRRIAAGGGGGGAVNTTNNNGQGQGNGRRR
RCGRPPQAKEQHRIKQEEVRFKVYDVVDVKQQQMPRGGPRPRTRSQQLFYYNHHNNNFDQ
NQKKQHLPKHYAAAAAAAADGNPILPHHRPSTTAAATTVAATAVNVKPVDEDLYKIPPEL
LLQTSKRKKMLGFFSKCLAPPCKA

>Cc_CDP03960
MDEYYFNGRRGHVPAFGSWDCNDDLPFTQCFETARQTGLFRYSYSEDRDLYVAGDLYEND
VVAPAMIVVPRRRVKSTSYQRQGKEAKGGEAWVVCDCECGVNVKEPPSPLPPSPPPPAPK
AVDEDLYKISPELLRAQPRKKRGWGFFSICMQPPSCVR

>Gr_KJB10851
MDDYYRRSHVPAFGSWDWNNDLPFTQCFETVRQTGLLRYSYAEDRDLYVAGDLYENDVVT
PAMIVVPRKKKKVGESHVKEGKKQRWEVSEEKAAAATSPIVMAKPTPKPVDEDLYKISPD
LLYPKPKKKKGLGLLSSCLVPSCLS

>Gr_KJB84110

MDDYYKRSHVPAFGSWDWNNDLPFTQCFETARPTGMLRYSYSEDRDLYVAGDLYENDVVT
PAMIVVPRRRNKVGQTHSKEGKNQRREVSDGKEAPEPRPRPRRKPVDEDLYKISPDLLYA
KPKKKRGLSFFSSCLLPTCVS

>Gr_KJB42592
MDDYYTRSHVPAFGSWDWNNDLPFTQCFESARQAGLLRYSYSEDRDLYVAGDLYDNDVVT
PAMIVVPRRRKKGRKSNDKEGKRQNWEINDVKESPSPTPLHRPTPKPVDEDLYKISPHLL
YAKPRKKRGLSFFASCMVPTCVL

>Gr_KJB44732
MDEYCKRSGQIPAFGDWDNANELPITQYFECARQAGLIRFSSSSGESNPYVAAHDLYETA
DSRKHSRNLAPPRKQASRVREKGGAHVKEQKKAGRVCDVTEPPRKYYQHPHHHVSLSVNN
DNTTSSNSKHPIYDAVPPPNPKRLPVRPPKPVDEDLYKIPPELLHSSKRKKMPGFFSCLV
PACAT

>Gr_KJB70634
MDDYNCKRSGQIPAFGDWDHANELPITQYFESAREAGLIRFSSSSAKPKPYLTVDLKTKH
PRNHVPVRKVS

>Gr_KJB80860
MDDYNCKRSGQIPAFGDWDHANELPITQYFESAREAGLIRFSSSTAKPKPYLTDDLKTKH
PRDHVPVRKVSRVREKREGSGGGGPLVTEEKKAGRVGGVTDPPRKLHYQYHHRHPHHHVP
NNINDCSKQHGNDVVPHKRLPVDEDLYKITPEILHSSKPVSAFSISFL

>Gr_KJB82497
MDDYNCKRSGQIPAFGDWDHANEFPITQYFESAREAGLIRFNSSSAKPKPYLTVDLKTKH
PRNHVPVRKDERFD

>Gm_KRH46588
MEEWYYKKSQVPAFGSWDWNDNLPFTQCFESARQAGLLRCSNYSESEERDLYVTGDLYEN
NVVTPAMIVVPRRRANVVDQHEKETKLKNWISDDVDSESPSPTPLPRPTSKPVDEDLYKI
SPGLVYAKAKKKRGLCFFSSCLLPTCIA

>Gm_KRH17687
MEEYYYQRHHVPAFGSWDWNDNLPFTQCFESARQAGLLRYGYSESEDRDLYVTGDLYEND
FVKPAVIVVPRRREKVRCQNEKDEKKQNWVSNVKELPSPTSPIQRPKPKPVDEDLYKISP
ELLYAKNRKKRGLCFFPSCLMPTCIA

>Gm_KRH66197
MEAYYYKRHHVPAFGSWDWNDNLPFTQCFETARQAGLLRYSYSESEDRDLYVTGDLYEND
VVTPAMIVVPRRREKVRCQNEKEAKKQSWRSNVKELPSPTSPIQRPKPKPVDEDLYKISP
ELLYAKTRKVLLFSPHTIIPFCFYLH

>Gm_KRG99533
MEECHYKKSQVPAFGSWDWNDNLHFTQCFESARQAGLLRCSYSESEERDLYVTGDLYENN
VVTPAMIVVPRRRAKVVDQHEKETKLKNWISDDVDSEPPSPTPLPRPTSKPVDEDLYKIS
PGLLYAKAKKKRGLCFFSSCLLPTCVA

>Gm_KRG92592
MDEWKRDGHNIPAFGNWDFTDEFPITCYFECATPARLHNKPRPLRNHIKQETRNKERRCR
HVNGNANKGKLYDVREQQRKPIRISKHVQVQQYDTVPRTPKPVDEDLYKIPPELLHTTNK
RKKLLGFISKCLVLLVCHEHC

>Gm_KRH68832
MDRSGQIPAFGNWDYANELPITQYFESARQAGLVRYSSSSGESDPYVRADRDLYAVDFKK
PIRNIPPSTLKKATRNRVVKEREKENVKMNMRKQGKVCDVTEQARKPVAAMHLDDAVPRL
PKPVDEDLYKIPPELLGTTKRKKKMLGFISKCLS

>Gm_KRH34194
MDEWKGDEHIPAFGNWDFTNEFPITRYFECATQSRLHNKPRHLRNHIKQETRNKERRCRH
VNGNANKGKVYDVREQQRKPNRISKHVQVQVQQHDTVPRTPKPVDEDLYKIPPELLHTTN
KRKKLLGFISKCLVLLVCHEHSKLKQ

>Gm_KRG97089
MDRSGQIPAFGNWDYANELPITQYFESARQAGLVRYSSSSGESDPYVRADRDLYAVDFKK
PPIRNMPPSTLKKATRNRGRRCVVVKEKEKEKESVKMNMRKQGKVCDVTEQARKPVTVSK
EMMRLHDAVPRSPLPRGVRLPKPVDEDLYKIPPELLRTTKRVRHRLT

>Mt_AET01348
MDHEYHFKRNHVPAFGSWDWNDNLPFTQCFESARQGGFLHYSYSESNEDQDLYVAGDLYD
NHVVTPAMIVVPRRREKVRSQHEKDVKKQNWVSHVVKEPPSPTPMSRPTPKPVDEDLYKI
SPDLLYVKTRKKRGLCFFQSCFTCIA

>Mt_AES82722
MDRSGQIPAFGNWDFANELPITQYFETARQAGLIRYSSSSGESDPYVHVRAEHHDLYAVE
YKKPVRKATRNRERKYQNVMVNEKETTMNMRKQQGKVYDVMEHPRKQMNKNKKVHVSAPL
QPTRLPKPVDEDLYKIPPELLRTTKRKKMLGFISKCLVPAACVS

>Mt_AES79021
MEEYYYKRSHVPKFGSWDWNDHNNFPYTNCFDSVTQPGSLRYSYSAESEDRDLYGTADFY
DNHVVSSTMLLNVPRKRAKVTDKHEKEIKKKNWVSKMDIELKPNPQPNLIPTSLPIEEDL
YNISPHLPYAKVKKRRGLCFFSTCFLPACIA

>Na_OIT36562
MEMECKRSGQIPVFGDWDNANEMPITQYFECARQAGLIRHSCHSSLEENNTDLYALHFHK
PHFYAIPNAPQRKTKAAINKKRCPEQKWTGKIEKLKNESGPTMKSATVAQRPTNPKPVDE
DLYKIPPHLLPGYKRKRMFGFFSRCLAPPCKA

>Na_OIS97516
MDESNYYFMRRSHVPAFGCWDCNDNEFPVPFTQCFESARQAGLLRYSYYEDRDLYVAGDL
YQNDIVTPTMIVVPRRKKKAVGKEGRKDCEYDDVKEAASPVPAAPRPSPKRAPKAVDEDL
YKISPTLLYAKPKRKGIKGFFSSCLRPICVS

>Na_OIS97515
LNPTSTIDSLQKANPFQASLIDPSLFYKFIVLVNKQKYCKFELSVVDCEVNLKGLFPDTP
LQKMDESNYYFMNRSHIPAFGSWDCNDDFPVPFTECFESARQADLFRYSYSNDRDLYVAG
DLYQNDIVTPAMIVVPRRKRKASTSNEVGGKKDAWVVRDYEYDVKKAATPSPPPPPPKRA
VDEDLYKISPELLYPKPKRKGVRGFFSMCLRPTCAS

>Na_OIT06648
MDESNYYLRRSHVPAFGSWDCNNNNEIPIPFTECFESARQAGLFHYSYSQDSDLYVTGDL
YQNDIVTPTMIVVPRPKKKASYKEARKEAWVMCDFEKEPPSPVPISPPTAISVPKKPIDE
DLYKISPELLYAKPTRKGVRAIISSCLRPACAC

>Na_OIT37923
MQQGSNYYFMRRSHVPAFGSWDCNDDSPIPFTQCFESARQADLLRCSYSEDRDLYVAGDL
YQNDIVTPIMIVVPRRKMKASNEAVGKAGKDTWVVCDYEYDVKEAATPTPPPPKRAVDED
LYKISPDLLYAKPKRIGIRGFFSSCLLPICAS

>Pt_PNT51591
MEDYYYSRSHVPAFGSWDWNNDMPFTQCFESARQAGLLRYSYSEDRDLYVAGDLYENDVV
TPTMIVVPRRREKMRRPRAKEEKREQSWAVTSDVKESPSPPPMSTRPTPKPVDEDLYKIS
PELLYAKTKKRGGLCFFSSCLMPACAL

>Pt_PNT00313
MAEWRRSGQIPAFGNWDQANDLPITLYFESARQAGLIQHSTNSSGECVHRYMRSDLHASD
FNKPSRYHVPPRK

>Pt_PNT06812
MDEWRRSGQIPAFGNWDQANDLPITLYFESARQAGLIRHSTNSSGECGHRYMRSDLHASD
FNKPSRYHVPPRKTRMREQRGPHSKEQRKQGKVCDVTEPARKQQPTMLHCHKIDAVICPK
VPLKPPKAVDEDLYKIPPELLRSAKRKKCPGLFSCLVPACAS

>Pt_PNS90127
MDEWRRSGQIPAFGNWDQANELPITQYFESARQAGLIRFSTTHNSSGECGHQDMRGDLYA
SDINKPSRNLPPPVKTRMREKRGTHAKEQRKQGKVCDVTEPARKQQQQPQPTVYHKNKIS
QYSHKMDTVIVAKAPVKPPKAIDEDLYKISPELLRSSKRKKRPGLFSCLVPACVS

>Sl_Solyc04g050790.3.1
MVMIEMESCRRSSGVQIPVFGDWDKANELPITQYFESARQAGLIHHSSSQQLYFHKTQFY
AIPNNLPHTKTKGAAINKKRCGGKVEKTSGPQRQTAQRPTNPKPVDEDLYKIPPHLLPGY
KRKRMFGFFSRCLAPPCKA

>Sl_Solyc06g083080.3.1
MEDNRYYMKRNQVPAFGSWDCNDNDFPIPFTECFESARQAGLIHYTYSQDSDLYVTGDLY
QNHIVTPTMIVVPRRKKKGSYKEGRKEEWVMCDNCEKESPRHVVPATTRHVHRKPVDEDL
YKISPDLLYTKSKMKGIRGFISSCLMPSCGC

>St_PGSC0003DMT400011483
CKRSSGVQIPVFGDWDKANELPITQYFESARQAGLIPHSSSQQLYFHKTQFYAIPNNVPH
TKIGGVSKFS

>St_PGSC0003DMT400016876
MDESNYYFMNRSHIPAFGSWDCNNDFPIPFTQCFESARQAGLLRYSYSDDRDLYVAGDLY
QNDILTPAMIVVPRRKRKSSITNEGVGKARKEGWVVCNCEYDEEKKDTSTVPPSPPPPKR
APKAVDEDLYKISPQLLHSKPTRKGVRGFFSSCLLPTCAS

>St_PGSC0003DMT400051990
MEETRYYLKRSQVPAFGSWDCNDNDFPIPFTECFESARQAGLIHYTYSQDSDLYVTGDLY
QNHVVTPTMIVVPRRKNKGSYKEGRKEGWVMCDNCEKESPRHVSVPLSPVPTAKHVHRKP
VDEDLYKISPDLLYPRSKMKGIRGFISSCLMPSCGC

>Tc_Tc01v2_p017730.1
MPLLPYFTLCFLSLIASPSPILNCQNSHAHPRKKQEKENTNWKLSSPSLLSFKGPLDLES
QVNLQDIPPLLSLSLSLSLCHNMDDFYRRSHVPAFGSWDWNNDLPFTQCFESARQAGLLR
YSYSEDRDLYVAGDLYENDVVTPAMIVVPRRRTKVRQSHVKEGKKQNWEVSDVKEPASPT
PLPRPTPKPVDEDLYKISPELLYAKPKKKRGLGFFSSCLVPTCAL

>Tc_Tc05v2_p021760.1
MDDYCKRSGQIPAFGDWDYANELPITQYFECARQAGLIRFSSSSGESNPYVPADLYAVDS
RKHSRNLDPPRKQVSRVREKRGLHVKEQKKAGRVCDVTEPPRKYQHQHPHHVPTSNNNNN
NSGKQLKNDAAPPRRPPKPVDEDLYKIPPELLHSSKRKKMPGFFSCLVPACAT

Short one-NOI monocot

>Bd_KQK01523
MSMEAGRTIPKFGAWDVNNPSSADGFTVIFGKARDEKKAPATVPGPGNVQYNKINRPGDD
IKDARAEKMNPSYNNNNANNARTNGSKKWFCCVSPSPTQP

>Bd_KQK18573

MSEESGRPLPKFGEWDVNDPASADGFTVIFNKARDEKKAGNGQDTESPCKDTRTERVESY
AAKTNSKWFCCVTPSPTQS

>Bd_PNT61875
MATNRGRPLPKFGEWDVKNPASAEGFTVIFQKARDDKKTTGPGQSGIPPAFRNNYNDGGS
SRSGFKSGKSYQYTRVPPTPRRVKKKWFFCCGC

>Bd_KQK23427
MENLKEGQGAAGVNAWMTVPAFGDWDMKNGAMPDYSMDFSKIREMRKQNKKELSRASLGG
DEDLLAQSQQQQQQQKPAKAQQPKLGRPANDHRRPLHGRDDSPTGRKRFLSYFQCCIKA

>Bd_PNT67356
MRRGPAFPAAWDPSPSLITTTLTRETSRKALLDLRLQIAVSLGSAFLIAAHGRPGSNKKA
SAPRRSSSGPEREREIPARRMASMETRKEEQGAAVGWMTVPTFGDWDMKNGPVPDYSMDF
SKIREMRKQNKRELSRASLGGDDDLLAQQQKATQQPQLQPGKAGFASSAADDPHHADQDD
SPTGRKKFLSYFQCCTRA

>Bd_KQJ87894
MEKIMEQEGKQKQQQERRSSVPAFGEWEETMKAAGGALPDYSLDFTKIRAARMQRKDAPL
SATWPAVHELSASGDRRSVGSDTDAGRHRRQHSDGTDLRRPLRPDRAAPKGRSKSKGCLF
GCMGGW

>Sb_EER89626
MAEESGRPLPKFGEWDVNDPASADGFTVIFNKARDEKKGGNGQDTDSPSKDTRTERVESY
AAKPSTKKWFCCVTASPTQS

>Sb_KXG30852
MSVAKDDTGRTIPKFGEWDVNNPASADGFTVIFSKARDEKKAPTTKPQPGHITQRSASAD
SKDSRTDKMTSYNSRTNASKKWFCCVSPSPTQS

>Sb_EES12880
MATPDKGQGRPLPKFGEWDVKNPATSEGFTVIFQKARDGKKTTGGPGNVRAGIPPAFRNG
DGADDGGYRPDFKYGDSSQHTPPKRIKKKWAFCGC

>Sb_EER88131
MEMEKMMGCQIPAFGMWNYCNDLSITQYFDSAMQARLMKRCWNRRGSDAAAAAAAVVVGE
KAGVACGDEQLVLFRTPSFQRKPAAQIKVIRREVEKHCDGNESQDGGGVQADEVVAYSVK
RKVIISKAVDEDLYKVPQPPMYQKPRKMRKVVWSMWIGCLGLDCVA

>Sb_KXG38577
MENRKEEQQGAAVGWMTVPAFGDWDVKNGAVPDYSMDFSKIREMRKQNKRELSRASLGGD
EDLLQQQQQQQSSKAQPPKSSAVVADDHHRRPLHDDSPTGRKKFLSYFHCCIRA

>Sb_EER92785
MENLKEQRGHDAAGGNNAWMTVPAFGDWDMKNGALPDYSMDFSKIREMRKQNKKELSRAS
IGGDDDLLAHAQAHKPQSNNAQPKLGRPRPADDHRRRPLHARDHSPTGGKKFLSYFQCCI
KA

>Sb_KXG33973
MEAQDDCKWRQIPAFGDWNLWDDMPVTQYFQAGTFFFAAPAEKDDEDLFKVPQFPAKPYS
YKKCVVRVKGEKETAVPPARKGGRRRYVNEQQKWKPKGAVVDEDLYKISPQLLCKVKKKK
LLRNLLGGCLGLSCIA

>Sb_KXG23466
MEQGQGGRRRQIPAFGRWNQHSDDDDVPITHYFESAVQAGLLVRPGGHCYHAAAAGELVL
FRSSASPPPHKPAKKVRSTTTMESNGHHQQLEAGSRRQPQRQEAFVAGDGGSRAAARPRR
PRVVRAAVDEDLYKVPSDMLRKKKGRKHVRSMWMGCVGLNCVA

>Sb_KXG28458
MKKKATARRQRIPAFGEWNYAYVGAGDWPVTQYFDSAMQAGRLVMAIPPPSPPKPAANKW
RESSGTLELEDEDEDEDEKQRQQHVVAVGHGGAVKKQGKQQQQQQSPVAHAYSKACRRVG
AAAAVVKAMDQDLYHIPPDMLCHEPRKRVTRRSLWMGCLGLDCVA

>Hv_HORVU6Hr1G050540.1
SGLKMEAGRTIPKFGAWDVNNPASADGFTVIFSKARDDKKAPVNVHKANRSADSKDARPD
TKKNTYNNSRANTLVSTNLHRR

>Hv_HORVU6Hr1G072080.1
MSMEAGRTIPKFGAWDVNNPASADGFTVIFSKARDDKKAPVNAHKANRSADSKDARPDTK
MNTYNNSRKKWFCCVSPSPTQP

>Hv_HORVU7Hr1G043550.3
PTKLSPKPLSLPLGGSDRRRAQSMSEESGRPLPKFGEWDVNDPASADGFTVIFNKARDEK
KAGNGQDTESPCKDARTERVESYATKANSKKWFCCVTPSPTQS

>Hv_HORVU2Hr1G103590.1
VICRAQNKGRSLPKFGEWDVKNPATADGFTVIFQKARDDKKTTAGPGKSGVPPAFRNTDG
GYGSVKSGNSYKYTRVATPKRAKVHTVRRSAARV

>Hv_HORVU2Hr1G068630.2
MQSNSSAKPRNRTAGMHTPERRASSEAQGQHTPGRGRTRQSNQSYNADDEVAVPPFGEWD
EANAESGEKYTGIFNRVRDDKLSPDSSARQQSSGNRRDENKVQQTCPCCIL

>Hv_HORVU4Hr1G075110.3
PMRACVQGEGATAWMTVPAFGDWDMKNGAMPDYSMDFSKIREMRKQNKKELSRASLGGDD
DLLAHHKQQQQQQQPSKAPQPTKLGAPAKDHGPLHGRDDSPTVSSLARSSPLLPAAY

>Hv_HORVU3Hr1G044700.3
GRFRWVCACVCGGGGATTWMTVLAFGEWDMKNGAMPDYSMDLSKIREMHKKNKKELSRAS
LSGDDDLLAHHGGSKVAAVEQEAKRVAAYDYEADPRWADYWSNVLVPPHLAACPDVLAH

>Hv_HORVU2Hr1G071440.3
MEKESHKEGEGATAWMMVPAFGDWDMKNGAMPNYSMDFSKIRKMRKQNKKELSRASLGGD
DDLLAHHKQQQQQPSKPPSPRRWPLMPGTLETRAIEGARDAADSAG

>Hv_HORVU5Hr1G029200.5
MTVPAFGDWDMKNDAMPDYSMDFSKIREMHKQNKKELSRASLSDDDDLLTHHGGPEVVAT
EEEAKRVVAAAYDYEADPRWADYWSDVLVSPHLATRPGVLAHFRCKFYQSFTVSTLTH

>Hv_HORVU5Hr1G046790.1
RQRQREARELKGEGATAWMAVLAFGDWDMKNGAMPDYSMDFSKIREMHKQNKEELSRASL
SGNDDLLAHHGGPEVAAAEQEEKQAAAAAYDYEADLAGPTTGPTSSSRRTSTPAQTSSP

>Hv_HORVU0Hr1G012080.1
RREARELKGEGATTWMTVPAFSDWHMKNDAMPDYSMDFTKIREMHKQNKKELSRASLSGD
DDLLAHHEGPEVAAAEQEAKRAVAVAYDYAADQRWADYWSNVLVPLHLAARPTSSPTSAT
SSARDSS

>Hv_HORVU2Hr1G087230.1
WREARELKGEGATAWMTVPTFGDWDMKNGAIPDYSMDFSKIREMYKQNKKELSRASLIGD
DDLLAHHK

>Hv_HORVU6Hr1G039070.1
RREARELKGEGETTWMTVSMFGDWDMQNGAIPDYSMDFSKIWEMYKQNKKELSRASLIGD

DDLLAHHK

>Hv_HORVU1Hr1G071050.1
RKIWIPNNMGGTTTWMTVPAFGDWDMKNGAMPDYSMDFSKIREMHKQNKKELSRTSLSGD
DRRPKRPAAAAYDYEADPCWADYWSQMSSSRRTSSPAPTSSCTSAASSTTDSS

>Hv_HORVU4Hr1G007060.4
AEGGEGAQGGGTTTWMTVPAFGDWDMKNGAMPDYSMDFSKIREMHKQNKKELSRTSLSGD
DGGEAAGGGGLRLRGRPVLGRLLVQCPRPAAPPRPPRRPRALPPQVLPQIHREHPQPSTP
SLRFIDC

>Hv_HORVU3Hr1G059370.1
AEGGEGAQGGGATTWMTVPTFGGWDMKNGAMPDYSMDFSKIREMHKQNKKELSRASLSGD
DDLLAHHK

>Hv_HORVU7Hr1G082470.3
PVCAGCHPTTECPHPRWWYKSANPPPPFSATDPLATTTPDLINPSKDLLAHARTMDMEME
RMMGCQIPAFGVWNYCSDLPITQYFDLAMQARLLKRHRRCCDAGERRLVLFGASPSPRKP
PQIKVIRRDVGEKQSDGGELLRELDGGVTGRAAAGSATKRVPAAGAVDEDLYKVPRPLMY
QKPRKVRGCLQTALL

>Hv_HORVU3Hr1G092940.4
MEAQDDCGKWRQIPAFGDWNMWEEMPVTQYFEPAATFFFTAQAGEDDVDLFKVPHFAANP
YTYKKVRHTQTQQNHSSSISRSA

>Hv_HORVU0Hr1G016170.2
IMTITRRGAGDCPPPGLRPHPHAAPAPRAVASPPRHAPPPANERHGAPQARAAKAPSAVP
KFGVWDEQNANANAAAQGFTVQFEKVKRHREAAKAAAPDVPPQLSPDRAAPTWGHPRRKP
KKSFLSKVYGCLFPVVRQ

Short one-NOI PAS

>Atr_ERN13743
MVVTTRFLGYQQDKGVPLPKFGEWDVNNPASAEGFTVIFNKARDEKKTGGNSGTSESPRK
SETSFKQEASYPSKSGVSHEYISFNLAFVPYTNYYS

>Atr_ERN19234
MTDKGGPLPKFGEWNVNDPASAERFTVIFNKARDEKKTGGNPRGPESPGKEHGSFAKSNS
NSIDHGASAENHGRDGSN

>Atr_ERN05607
MDKGRPLPKFGEWAVNDAASAEGFTVIFNKGRDEKKTGGNPCGPESPGQDDGAYKHGSFA
KSNSVSVSFHRMNSHRVMIDIVLERT

>Atr_ERN06768
MEEYRRGQIPAFGNWDGGESLPITQYFESAREAGLLRRHAEENMSGFRQTIKSGEVMQKP
RTKPHQHTKDQKKKVGVRVCSVSEPMPRKPAVTPKPVDEDLYKIPPELLRRSRRVGRART

>Atr_ERM94361
MEDRKERNACVSVPQFGAWESQCDMPNYSMDFSMIRELKKQNKKDFSRASLGNEEELIHH
HHHSNSPTTRKKLLSYFNCFIKA

>Sm_EFJ13336
EKAAVLPKFGAWDANDPASGDGFTMIFTNARNEKKAGGSVHVPPLGVDEKDLYPERRTSA
SQKTSTVCMDLLFSFYGLI

>Sm_EFJ24675
EKAAVLPKFGAWDANDPASGDGFTMIFTNARNEKKAGGSVHVPPLGVDEKDLYPERRTSA
SQKTTSTVCMDLLFSFYGLI

>Sm_EFJ23521
GPPLPKFGAWDPKDPSSADGFTIIFNKARDEKRAGSGGRPASPVKNDNELYKNNPDRSSS
NVMQSSLFPLPSR

>Sm_EFJ34808
GPPLPKFGAWDPKDPSSADGFTIIFNKARDEKRAGSGGRPASPVKNDSELYKNNPDRSSS
NVMQSSLFPLPSRIADRVLMQKKWYCCFGAAET

>Sm_EFJ37612
ERNAPALPKFGDWDPKDPSSGEGFTTIFNNARNERQPGRIQQDSAPDHQQHGYGRGGGAH
PGRKQQVSPRDSIGEKRNSLTVSILSSLQSNWFCCFG

>Sm_EFJ15079
ERNAPALPKFGDWDPKDPSSGEGFTTIFNNARNERQPGRIQQDSAPDHQQQGYGRGGGHP
GRKQQVSPRDSIGKKRNSLTVSILSSFQSNWFCCFG

>Pp_PAC:32967352.CDS.1
MAKKGNIVVTRPREVEKSFVLKPGEGLNNCNLITHKVIHVDQSPRPSSVQIPKSSGNEPK
SSKYASPMARARQETKRRGGGESLGNSKDWPNLSDMDSSSNPPSESGISVGDTPSRPVPY
SGYIAKPRGSVSTADGEDSAGNSRLPKFGVWDNNNESSGPCYTLLFQNASQEKKVGGPVR
IHVPPPCSPAQEGDLYSYHSGMAKSKRKNQCSLLCCFAMR

>Pp_PAC:32925459.CDS.1
MAISLSVVDDKTNKVERNMVLESGEDARRTDSNAEKLAVATKPTEHQIPKAQSPGIPKRR
TPPRSGKKYVSQMAQAREEKKRREASSGKSKDWPGSSDADTSSTPSSADSGSLTSDVPNG
VQREGAAAVAKNGGSTASKLEAEKAVGGPGHLPKFGEWDNHTVDSGPCYTLLFQNAAEQK
KNGGPVLVHAQKPSSKPGVTEDLYDFNYGGMKSKKKKQFILLCCFSSA

>Pp_PAC:32958873.CDS.1
MCCGLHNLLTMTVSQSVVADKVVDLEKDIVFKSIEHYIAQSLPEPPRTSAFETPRASSAE
ASKPRDTLRAGWKDRPAKYVSPMAKARAEKKCKEISSGKSKDWPTLSDMETSSTPSSGED
GSLHLELSNGLQTKAPSVGVKARPSTASKEDAEKVLGGSGHLPKFGEWDKHSANSGPCYT
LLFQSAAELKKTGGPVRVYAQKPPSSPGVTEDLYDYKPVSAGARAKKKTQCGFLSCFSS

>Pp_PAC:32937320.CDS.1
MDKLRNILMARARDAEKSSVQKPGEGSKSCDVLQSDQSPRSPSGKECTFSKHVSSKVQSY
QETKRHGLGESNEEFKDWPKLSEMEMSSNPSTKSALSITNTSPWPVPNSGYISRHRGHAS
TAEGEVGGRRGHLPKFGEWDNQDDAYDPCFTLLFQIVSDEKKGGVPILVPVPQPSTSARE
GDLYSYHSGLAKSKRKSLFCLLCCFAVR

Long two-NOI dicot

>At_AT3G25070.1
MARSNVPKFGNWEAEENVPYTAYFDKARKTRAPGSKIMNPNDPEYNSDSQSQAPPHPPSSRTKPEQ
VDTVRRSREHMRSREESELKQFGDAGGSSNEAANKRQGRASQNNSYDNKSPLHKNSYDGTGKSRP
KPTNLRADESPEKVTVVPKFGDWDENNPSSADGYTHIFNKVREERSSGANVSGSSRTPTHQSSRNPN
NTSSCCCFGFGGK

>At_AT3G07195.1
MANRPHVPKFGDWNNQDQPFTVVFDNARTNKRQDLYESIEKPETKPQELAPPPPQPARRIQKPEAP
KPVKQDTPRAPPPTEKNRVKAPPADQLYGGGGSGGGGRSGSGSAGGLYGGYGGGSVGNQRQPPAP

RPTQPRQNLRGGNNGRGGTTIPPFPGSVGAGGDMSYTQIFEKVKEERNEGVIRPNGGTAGNTPSRPI
NSQHDQSTNQTSSKGCCFSWCRRGSKY

>At_AT5G48657.2
MANRPHVPKFGDWTEDAPFTVVFEKASKSKKNMNVANPNEYPDMNPNAAQNRNMSRPDQQPPN
HNVRPRHERFNSRDETEFRPSPAHNERNNRVRSVPPTPETYNHQTYGGGGRSMGNPTEINRRQSRD
HVPARPIRNLRGQSSERVATIPPFPGTGSNMENQSYTLIFDKVKEDRNQARSYNGTDHSTPTRPIIDQ
HHQPLPSSPKGCCFPPWSRKGS

>Al_fgenesh2_kg.3__2715__AT3G25070.1
MARSNVPKFGNWEAEENVPYTAYFDKARKTRAPGGKIINPNDPEYNSDSQSQAPPPPSRTKAEQVE
PVRRSREHMRSREESELKQFGDAGGSSNEAANKRQGRASQNNSYDKSPLHKNSYDGTGKSRPKPA
NLRADESPEKVTVVPKFGDWDENNPSSADGYTHIFNKVREERSSGANVSGSSRTPTHPNSSKPNNTS
TCCCFGFGGK

>Al_fgenesh2_kg.8__747__AT5G48657.2
MANRPHVPKFGDWTEDAPAFTVVFEKVSKSKKNMNVSNPNEYPEMNPNAAQNRNMSRHDQQPP
NHNVRPRHGRFNSREETEFRPSPAHNERNKRVRSVPPTPETYNNQSYGGGGRSMGNPSETNRRQSR
DHDPVRPIRNLRGQSSERVATIPPFPGTGSNMENQSYTLIFDKVKEDRNHARSSNGTDHSTPTRPIIN
QHHQPLPSSPKGCCFPPWNRKGS

>Al_scaffold_300847.1
MANRPHVPKFGDWNNQDQPFTVVFDNARTNKRQDLYESIEKPETKPQEQAPPPPQPASRNQRPEPP
KPVKEDTPRAPPPAEKNRVRAPPADQLYGGGGSGGAGRSGSGGGGGLYGGYGGGSVGNQRQPPA
PRPAQPRQNLRGGNNGRGGTTIPPFPGSLGAGENMSYTHIFDKVKEERNE
GVRPYGGTAGNTPSRPINSQHDPSPKQTSSSMFCCFSWCRKGSKY

>Ah_g28247.t1
MARSNVPKFGNWEAEENVPYTAYFDKARKTRAPGGKIINPNDPEYNSDSQSQAPPPPSRTKAEQVE
PVRRSREHTRSREESELKQFGDAGGSSNEAANKRQGRASQNNSYDKSPLHKNSYDGTGKSRPKPAN
LRADESPEKVTVVPKFGDWDENNPSSADGYTHIFNKVREERSSGANVSGSSRTPTHPNSSKPNNTST
CCCFGFGGK

>Ah_g11847.t1
MANRPHVPKFGDWNNQDQPFTVVFDNARTNKRQDLYESIEKPETKPQEQAPPPPQPARRNQRPEPP
KPVKEDTPRAPPPAEKNRVRAPPADQLYEGGGSGGAGRSGSGGGGGLYGGYGGGSVGNQRQPPAP
RPAQPRQNLRGGNNGRGGTTIPPFPGSLGAGENMSYTHIFDKVKEERNEGVRPYGGTAGNTPSRPIN
SQHDPSPKQTSSMFCCFSWCRKGSKY

>Ah_g15965.t1
MANRPHVPKFGDWTEDAPAFTVVFEKVSKSKKDMNVSNPNEYPEMNPNAAQNRNMSRHDQQPP
NHHVRPRHERFNSREETEFRPSPIHNERNKRVRSVPPTPETSNHQSYGGGGRSMGNPSETNRRQSRD
HVPVRPIRNLRGQSSERVATIPPFPGSGSNMEDQSYTLIFDKVKEDRNQARSSNGTDHSTPTQPMINQ
HHQPLPSSPKGCCFPPWSRKGS

>Br_Bra013233.1
MAERSNVPKFGNWEGEENVPYTAYFEKARKGRAPGGRVTNPNDPEYNSDSQSQAPSRTRPEEVDP
VRKSREVTRSREESELKQFGGGGGDGSGSSNEKRQGRSSQNNSYDKSPLHKNSYDGTGRTKPKPNL
RADESPEKVTVVPKFGDWDENNPASADGYTHIFNKVREERSTGANNVSGSSRTPTHPNSRNSPSSSS
KCCCFGFGGK

>Br_Bra001241.1-P
MTNRPHVPKFGDWSNQDQPFTVVFDNARTNKRADLYESLENSDIKTPPQPAPRIPRPEPP
KPAREGTPRAPPPTERNKVRAPPADQLYGGGRDGGGLYGGYAGGGGSGNRQQQAPPRPAQ
TQPRPNHRGGSNGRGGTTIPPFPGSVGSGENMSYTHIFDQVKEERREGARPYGGTAGNTP
SRPINSQHESPSPNSSKVCCFPWGRKGSKY

>Br_Bra036163.1-P
MAANRPHVPKFGDWKEDVPFTVVFDKASRTKQNANMSNPNEYPNMNPSPAQTPNHRYDQP
PNHNARPRHERFSSREETEFRSSPAHNERNNRVKAPPPAETYNHQAYGGGGRSHGNPFEP
NRRQLHEPPRVQPIPNLRGRNSERVAIPPFPGSGSENQSYTLIFDKVKEDRRQSGNVRSY
NGSSHTTPTRPLNDQRHQPLPSSPKSCCFPPWGRK

>Br_Bra037470.1
MAANRQSLPKFGEWTEDVPFTVVFDKASRSSRKNTNKSNRNPNEYPEMNPTAAQTRNQRH
DQPPNHNVRPSQERFDRREETEFRPSPAYSERNNRVRAPPPAETYDHQSYGGGGTNPSET
NRRQPYDHTPVKPRPISNLRGRGSERVATIPPFPGSGSEDQSYTLIFEKVKENKRQSGTV
SSYDETDHSNPTPLINDDQHHQPLPSSPKGCCFPRWCRK

>Mt_KEH18185
MAQRTHVPKFGNWESNDNVPYTAYFDKARKGRTGTKMINPNDPEENVDLVLDNSSSDHPP
PSNTKPRANSEDLSGKGLVRSTIESHKSLDGDPKQYVDSPARHDNASNRSSNDSTPRLGV
GSADNRRRPSRQSTAGSEHSVERSPLHRQARAPAGRDSPSWEGKNNSYDSSHGTPGRSRL
RPANRGDETPDKGAAVPKFGDWDVSNPASADGYTHIFNKVREERQGGAGHAPGTPNERPH
VIRNQNNDKAQCCCFAWGKK

>Mt_KEH22625
MTTQRSHVPKFGNWEGEDDVPYTVYFDKARKSRPGSKMINPNDPEENPDLVLQNSSSDDV
IPPKPRVSSENQSEKGTVRLTHNDLQKNKEEGDVKHSVNSPARPGGHGVGSADSRRRPSR
QSTASSEYSVERSPLHRQAKTPGRDSPSWEGKSTYDSSHGTPGRSRLRPVNRDDEIPDKS
AAVPKFGEWDESDPASADGYTHIFNKVREEKHVAAGNTPGTPNGRSYVIRNQPANDKAQG
CCFFWGRK

>Cc_CDP11146
MAQRSQVPKFGNWESEEDVPYTVYFDNARKGKKGSKMNTNDPQEDLDAETKGQKRPEATR
AKHVRHTSREDGDLRKSIDSPLHSDAMSQKSANESPHHKQGGLKHGSRKPESEGSKGTDT
VRPRHESREEGDLRRPTDSPLRNETGNRRTSHDSPHHRHGGLSAGETPKRVARQSVGSDR
SIDQSPLHPHSQVRTGGRGSGVSSPSWERKGSSEGGLGLAPSTPGGSRLKSVTRGDETPD
HSPAVPKFGDWDETDPASAEGYTHIFNKVREERHSGAGKVPGMPTESSYSNGQKPIGNDN
SKCCWCFPCGR

>Cc_CDP03897
MAARPHVPQFGNWDSQENVPYTLYFDNARKGRGGKMINPNDPEENPEMFRQFAPPAPAPS
NIRPQTEEPVGRGAVRAQHEYRGSREDGNFRQFVDSPARTDNPGRRTSGESTHHNSGRPT
RHSAGSEHSFERSPLHSHHPAKLAGRGGTSPAWDGKSSHDSSHGTYGKSRMKQVSRVEES
PDRGAAVPRFGDWDENNPQSADNYTHIFNKVREERHTGPSNLQAGNNFEPSYHMKRKQNA
SGDRKGCCFPWFGK

>Cc_CDP04463
MTWLQQRSHVPKFGNWEGENVPYTAYFDNARKDKISGTRMNPNDPEENPEAFVFSGLING
GEASEFSPTPLQRNLVRPISSEKYRQKQRYGHWRNTSDEPKSVGQKSPGSESITDKNNYD
NPHQQKRSERKKSMMTENSNSFIPLSPARRARGGSDTSDDLSYNSASVPKFGAWDERDPR
SGEGFTVIFNKVKEEKRIAASKFAPAQQPQSSNNAESHKRDAKSKVCNLFLQGNARNLDP
ARAFISL

>Tc_Tc01v2_p018410.1
MAQHSHVPKFGNWESEENVPYTAYFDKARKGRTGGKIVNPNDPQESPDLHYDYVAPTRAS
PASRAKPELDEPVGHGPTRRAHERGRSREEGELKQYADSPSHHDNVNRRASGDSTPSRYG
RGVNVGEAPKRPARSSIGSENSIDRSPLHHQARVGRGSMASPAWEGKNSYDSSHGTPGRS
RMRPSTRGDESPDKGAAVPKFGDWDENNPASADGYTHIFNKVREERNNGGRVPGMPGEQS
PYHTGQNRKPTNSSAKSCCLPWCRK

>Tc_Tc06v2_p019020.1
MDMSHVQLRSHVPKFGDWDNDNLPYTAYFENARKERAGIRMNPNDPEENPEAFMYTRGGL

ESNCDCQPVQVPPAAGSQKSIPADKNHNDGHSRQNAPNRNGSYDYQKSVRSHRSMASESG
SEKSNSDHSLLRFSHRRANSGQKKGRVGGSSFSSSVSGQFTHRNGSYQSDNNRHRRTASI
PKFGEWDETDPTSGEGYTAIFNQVKAEKQSPTSKFPTVPPQQSNYSDCYHSKPRTPSFCS
KVCCCLFSRGSD

>Pt_PNT51490
MAQRSHVPKFGNWESEENVPYTAYFDKARKGRTGGKMINPNDPQENPDLVSDYAAPDQAP
PFRAKAPPEEAAGQGAVRQAHEHRTSREESDLKQFANSPARNENLNRRASYEPAPQRYGG
RGPSFGEAHKRPARYSIGSENSMEQSPIHNHARISGRNSGAPSPSWEGKNSNDGSHGTPG
RSRLRPKGDESPDKGAAVPKFGDWDENNPSSADGYTHIFNKVREEKQIGEGKMPGMPTES
SNAYVRKQTPSDSAKCCCFPWGRN

>Pt_PNT11440
FLYVFKLNDPHPKLNSFDLHSKISQFFTSPAPSMAQRHHVPKFGGWDNDNVPYTAYFDTA
RKEKSGMRMNPNDPEENPEAFMQARGGMEDDVDFSSGAYQGITAEHHNIDQRLKGHNAHG
TRSANDHQKSASHNSITSESGSEKSSSEGNKALSSSRHSRQRSRSTHSTNHDGHQRGASI
PKFGAWDETDPNSGEGFTVVFNRVKEEKQIASTTFPSVPTQPVNRQTSQRNQGSSSSLSK
FCCCFFPRGK

>Pt_PNT38893
MSSHRLCQLCNNRIIVSNFAITAAKLVIILVLSFSSASSLNHFESSDPSMSQRSHVPKFG
GWDKDNVPYTAYFDNARKGKSGVRMNPNDPEENPEAFMYARGGMEDDVDFASGAFEGTNS
EKQNIEGLKGHNAHSMSPSDHRKNARHNNLTSESGSEKSSSDHSLLQAQKTGLAVDGSTH
PFDHEGHHRAASIPKFGAWDETDPRSGEGFTVIFNRVKEEKQIASTTFPSVPTQPVSLRN
KGNSSSRSKFCCCCFPKGEMND

>Na_OIT08311
MARPNVPKFGNWENDDNTPYTVYFEKARQTRGTGKIINPNDPEENPDMFPNLTPPPPAPS
RARPKAQTEEPIGHEGAAAKQTREHRLSKEDGDFRQYANSPARNENTGRRASNEHQRGRG
PSSGRTGRQSAGSEHSFDKSPLHPHYQAKVNAGRGVASPAWEGKNNSYDSSHGTPGRSFE
SAHATPGRSKIKQESPDRGAAVPRFGEWDENDPQSADNYTHIFNKVREERQLGTGNPSGA
PSRTSYNPQREEEKRMKCCFPW

>Na_OIT28923
MARPNVPKFGNWESEDNTPYTVFFDNARKTRGGKMINPNDPQENPDMFHNFAPKTQREEQ
KRRVSKEHGDIQQRNGAGRGSNSTRPARQTAGSDHNIAKSPIHPYSQQAKISGRVAASPA
WEGKNSYDSSHGTTPGRSFESARATPGRHQLKKESQPDRGAVVPKFGGWDENDPQSAENY
SEVFNKVRNERNERNRGSGNVLGTPGRTSYSTERHQRNEKQKSCCFPLW

>Na_OIS96371
MAKHSQVPKFGNWENDEDVAYTTYFENASKGKKGSKMNPNDPQDPEAEVKGQNGRDAVRS
KDERFSRRDDVESRKSIDSPLHPDAMGQKAPTYSSPQRYGVKSVGNKSESETMKGAEIPT
ARHERRPSREEYLRRPTDSPMRNENTGRRTPMESPHHRYGGLSGGATPKRASQQSGGPDR
SIEHSPLHPHSQGRVGGKGGAVSSPSWERKVSSEGSQGLAPSTPGRSRLRSVTKGDDMSD
DSPAVPKFGDWDESDPASAEGYTQIFDKVREEKQTGAAKVPGTSTDTSYSNSQKRYGNDS
GKGCLCFPWGRS

>Na_OIT39574
TRRRIPKSLMAQGSHVPKFGNWDGDNVPYTAYFENARKGKGGKMINPNDPEENPEAFAYR
GYEDASINVSPAKTPLIETPQYNYGHRRNPSDDLSNYSSASVPKFGAWDEKDPKSGEGFT
VIFNKVKEEKQIAAAKFPIVQPQPNMPSSHKRNTKSKMFCCLF

>Na_OIS97631
FPKKIPPFSLFFFLSNFSSNMAKQREKYGLPYVPQFGAWDHKTGDNLNFSMVFSQARANK
KQNRHNLAQHNLGNEQEILAKLQEVSPREDSSTPVPQFGERNQKTEGNPDYSKVSPKAHA
NKKSHRHDLTHRSLGNEKELGKHQEVSSMKRPMSVPQFGVWDQKSGGSPDYAKVSPQARA
EKKQHKHASARRKLGNEQELRKHNEASPRKNSPVAMPQHGAQDQKTGDNPNYPMVFSQAH

AKKKQHKPHSLGNEQELGKRQDVSPMKTGWLSVPQFGEWDQKTPSETNYSVVFSQARANR
KHHKSDLTHRSYDFEQELLCREREQAARRKKNKFLTYLSCCLPV

>Gm_KRH66117
MAQRSHVPKFGNWDSGENVPYTAYFDKARKGRTGARIINPNDPEENADLSLDNPSSDHLPPTRPRA
NSEDQSGKGSLPLEDDPKHFVDSPARHDNVSSRSGSRSHGVGSAENRRRHSTQSTGSEYSIERSPLH
RQARAPGRDSPQWEPKNSYDNSQGTPGRSRLRPVNRGDETPDKGAAVPKFGDWDVNNPSSADGFT
HIFNKVREERQGVPGQVPGTPNERPQAIRGQSNDDKVQCCCFAWGGKK

>Gm_KRG99728
MAQRSNVPMLGKSEENVSDTAHSDKAQKGQPGSKMINPNDTKENSDVVSSAGLPHSKPRV
HSEDPSGKGSVRSIHELQMSREDGDPKQFTDSPARHGGSDSAYRGHGVGSADNRKRPSRQ
STGSEHSIDRSPLHRQAKTPGRDSPSWEGKNSYDSSHGTPGRSRLRPPNRGDETPDKGAA
VPKFGEWDESNPASADGYTHIFNKVREEKQVGAGHVPVTPNGRQYAARNQPADDKAQSCC
FCWGKK

>Gm_KRH46664
MSFVSFLCFFSVDNSEQHSNVPKFGNQESEDNVLDTAHSDKAQKGQSGSKMINPNDTKEN
SDIVSSADLPHSKPRVHSEDPSGKGSVRTTHELQKSREDGDPKQFTDSPARHGGGDSSHR
GHGVGSADNRKRPSRQSTGPEHNIDRSPLHRQAKTPGRDSPSWEGKNSYDSSHGTPGRSR
LRPSYRGDETPDEGAAVPKFGEWDESNPASADGYTHIFNKVREEKQVGAGHVPGTPNGRQ
YAARNQRANDKAQSCCFCWGKK

>Gm_KRH07458
MAQRSHVPKFGNWDSGENVPYTAYFDKARKGRTGTRIINPNDPEENADLSFDNPSSDNLP
PTRPRTNSEDQSGKGSLHLEDDPKNFIESPARHDNVSSRSGSRSHGVGSADNRRRHSTQS
TGSEYSIERSPLHRQARAPGRDSPQWEPKNSYDSSQGTPGRSRLRPANRGDETPDKGAAV
PKFGDWDVNNPASADGFTHIFNKVREERQGGPGQVPGTPNERPQPINGLSNDDKVQCCCF
AWGGKK

>Gm_KRH36523
MAQHSHHVPKFGNWDTDNVPYTSYFENARREKSGIMINPNDPMENPEAFNTCIRVDADEV
MMASHGYSHNVHSLENGSHVRRRSRGSNGELTVTAEFGASEQSHFDHSVNHKRNMSKGGS
SIKGFSSSSHNTQRTANSSFSDHSNHRATAIPKFGTWDVTNPKSGEGYTAIFSKIKEERQ
IKSSHVSSIHSTPPLNNSNIKNQYGESSSWLSKYCCCCFQAGQSK

>Gm_KRH11509
FGNWDPDNVPYTAYFEHARREKSGIMINPNDPMENPGALNTFMLMNGYSLENGSHVRPRS
RGSNGGLTVTAEFGSEQSHFDHSVTHRSPQSDHQRNMSKGGSSTKSFSSSSHNTHRSTNS
SFNDHANHRATAIPKFGIWDVTNPKLGEGYTAILSKIKEEREIKSSHVDSISSPPLNNSN
IKNQYGESSSWVG

>Gm_KRH02007
HSHVPKFGNWDCDNIPYTVYFDNARNKKPFINPNDPEQNPEVFNLYMRGVEKSDEAVKAS
STRMRMRSYSTSSLEQTSHEHDHEEGRSNTKSHSHHTTESVSERSNSDYSVIQRVKSDFI
GSFSSSNHNIKGRGGSHSLINDHVNHEAALVPEFGAWDVTDPKSGEGYTAIFSEIRKEKE
IASGRMPKSMWQTFLHFIQGNIKISLVGYKLIKY

>Gr_KJB10918
MAQRSHVPKFGNWESEGNVPYTAYFDKARQGRSGGKIVNPNDPQESPELHQDYGAPTRAP
PASRPKPESDEPIGHGPARRGHERGRSREEGDVRQYADSPARNENVNRRAAGDSTPSRYG
RGVSSGESQKRSTRTSIGSENSIDKSPLHPARGTGRGSMASPAWEGKTLNDSSHGTPGRS
KLRPSKGDESPDKAAAVPKFGDWDENNPSSADGYTHIFNQVREERNNGGRVSGMPGQQSP
YNTGRNRKPTNNSAKGCCFPFWRK

>Gr_KJB26120

MAQRSHVPKFGNWEGEEDVPYTAYFDKARKGRTGGKIVNPNDPQENPDLRRDYVAPNKAP
PAPRTKPEADELVGDGPVRRGHERGRSRDEGDFKQYAGSPVRHDNVNRRALGDSTPTRYG
REAPRQGTRPKVGSENSIEKSPLHHQARVAGRGTMASPAYEAKTLYDSSHGTPGRSKMRP
NTRSDESPDEGAAVPKFGDWDENNPASADGYTHIFNKVREERNNGGKTPGMPGEQSPYRT
TRNRKQANSNAKSCCLPWCRK

>Gr_KJB67308
MSEAKLRSHVPKFGDWDNGDLPYTTYFENARKEKAGIRMNPNDPEENPEALMYTRGGPES
NYDGRSVPVTADKHHQNAANKDGSSYDHQKSARRQRNTALESENNTGSDRSILPSNHRRR
NSGQKNGRPGGSAFSASVSGQSQRTGNHQLDGNKHHRTPSVPKFGEWDETDPTSGEGFTV
IFNRLKEKQAAPSSNYQTVAPEVKTMNYSKLKICCCLFSRGNE

>Sl_Solyc12g098440.2.1
MAKHSQVPKFGEWESDEDVQYTTYFENAAKGKKGSKMNPNDPQYLEAKVKGENGTDTVRQ
KPERIASRDDVELRKSTGSPMHPDTMGHKVPTYPSPQRHGAKYGGNKSESETMKSTEILT
PRHERRPSREEGYLRKPTDSPLRNENMGRRTPMESPHHRYGGLSGGATPKRASQQSVGPD
RSIEHSPLHPHSHGRPGGKGGVVSSPSWERKASSEGSHGLAPSTPGRSRLRPVAKGDDTP
DDSPAVPKFGDWDENDPASAEGYTQIFNKVREEKQTGSAKVPSSSTDTSYSNSQKRYGND
SGKGCLCFPWGRS

>Sl_Solyc09g059430.3.1
MARPNVPKFGNWENDDNTPYTVYFEKARQTRGTGKMMNPNDPEENPDMFRNLAPPPEVAP
QSKPKRQTEEPPIGRGGPARQTRDHRLSKEDGEFRQYANSPARKESVGRKGANEPSHQRG
RGSNSGRTGRQSIGSEHSFDKSPLHPHYQAKVSNAGRGVASPAWEGKNNSYDSSHGTPGR
SKVKQDKSDRGAAVPRFGEWDENDPQSADNYTHIFNKFREEKQGNPSGTPSRTSNNTQKH
NSEEKQRKWCCCPW

>Sl_Solyc06g083390.3.1
MARANVPKFGNWGNEDNTPYTVVFENARKNRGGKMINPNDPQENPDMFPNVAPSSRPKTP
PTEEPMGMETARQTNKRRVSKEDGDFRASSPARNEPTTHQRHGGGRGSNSGRPSRQSGGS
DHSIAKSPLHPNSQAKISGRVAASPVWEGKNLYDSSHGTPGRSFESSHATPGRHQMKQES
PDRGTVVPKFGGWDDNDPQDAENYTEVFNKVREQRHVDTGNMPAAGVRTSYSTQRQQRNE
KQK

>Sl_Solyc11g012010.2.1
MAGSHVPKFGNWDGENVPYTAYFENARKSNSKGGKMINPNDPEENPEAFAYCGDEDANIN
ISPLVEKHQYHYDHRRNPSVESGQNKSIGPTNSNSESFGDSQRKSVSGFSVNQPTRRRRT
SDVKKNKNDRGNGFVPPSPNRPMKNSRNPSDDLSCSSAASVPKFGAWDEKDPKSGEGFTV
IFNKVKEEKHIAAAKFPVVQPQSNMSSSNNHKKNAKSKVFCCLF

>Sl_Solyc11g064930.2.1
MAKERERNGSPSVPQFGAWHHKTADDLNFSMVFSQARANKKQSRQNIAHHNPGNEQEMLG
KHQDVSPRKSSSTPVPQVGPRDSKDRANKKPHKPDKTHRSLGNEQELGKHQQASTMVRPK
SVPQFGEWDQKSGGSPDYSKVSPQACANKKQHKHDSAHRSLGNEQELGKHREVSPRKNSH
TAEPHCGAWDQKTGNSPNYPMGLSHDRAKKKQHRHGLARHSMGTEQELGKHRDVSPVKIG
WMSVPQFGEWEQKTPSETNYSMVFSQARANRKKHKSDLTHRSYDFEQDLLSREREKAATR
KKKKFMTYLSCCLPA

>St_PGSC0003DMT400011621
MAKLSQVPKFGEWERDEDVQYTTYFENAAKGKKGSKMNPNDPQYLEAKVKGENGTDSVRQ
KPERIASRDDVELRKSTGSPMHPDTMGHKVPTYPSPQRHGEKYGGNKSESETIKGPEILT
PRHERRPSREEGYLRKPTDSPLRNENMGRRTPMESPHHRYGGLSGGATPKRASQQSVGPD
RSIEHSPLHPHSHGRPGGKGGVVSSPSWERKASSEGTHGLAPSTPGRSRLRSAAKGDDTP
DDSPAVPKFGDWDENDPASAEGYTQIFNKVREEKQTGSAKVPSTSTDTSYSNSQKRYGND
SGKGCLCFPWGRS

>St_PGSC0003DMT400071827

MARPNVPKFGNWENDDNTPYTVYFEKARQTRGTGKIMNPNDPEENPDMFPNLAPPPEVAP
QSKPKKQTEEPPIGRGGPARQTREHRLSKEDGEFRQYANSPARNENMGRKGANEPSHQRG
RGSNSGRTGRQSIGSEHSFDKSPLHPHYQAKVNNAGRGVASPAWEGKNNNSYDSSHGTPG
RSKVKQENQSDRGAAVPRFGEWDENDPQSADNYTHIFNKVREEKQGNPSGTPSRASNNTQ
KHNSEEKQMKWCCCPW

>St_PGSC0003DMT400051789
MARANVPKFGNWGNEDNTPYTVVFENARKTRGGKMINPNDPQENPDMFPNVAPPSRPKTP
TEEPMGMETVRQTNKHRVSREDGDFRASSPARNEPTTHQRHGGGRGSNSGRPSRPSGGSD
HSIAKSPLHPNSQVKISGRVAASPVWEGKNSNDSSHGTPGRSFESSHATPGRHQMKQESP
DRGTVVPKFGGWDDNDPQDAENYTEVFNKVREQRHVDTGNMPAAGGRTSYSTQRPQRNEK
QKV

Long two-NOI monocot

>Bd_KQJ99083
MAKQQQKNGAHVPKFGNWDNDGNVPYTLYFDNARKGKAPGAKPMNPNDPLENPDAFSSSY
AAAEAAAPPPPQPPAPAAHHERRPSSEVPAPAPPLYPGSPFHRGEPPRRVSGGGGRTSGG
GGGGAYSVEQSPSRSPLHPYSRAADYSEASGFGLVANSVDRPRPRRGNETPTRGSAVPKF
GDWDSNPASADGYTHIFNKVREEKQTQAKTPGFGKDGAAARGPGQHDDGYVSSSRWCFGW
CK

>Bd_KQK12122
MAEHKGVPKFGSWEDEGQGDHLYTQYFENARKGKSPGRSVNQNDRNEDTEALSNDPPSIK
ASPLRAGSDPGQRKQKDERRANREDDLRRHEATARKPYADSPNHRYGDPANYDGTARKAS
NERSPMHARHQTRLANKGGVSSPSGDRRGSAPTTPGRSKLRSSGRGDETPERGSAVPKFG
EWDEKDPSTGEGFTDIFDKVREEKQSGLDNVGTSNPYYMDRANGGRRNGSSGCSCFNWFK
N

>Bd_KQJ82214
MYLVKQQPRIPTFGDWENSEDTPYTQKFEGARKNKKTGIYSNPNDPGHQPEPPRRSPLNP
SSYTPDAREQGPRNPPHGRRPETDPHNREPVPRRHSTPQQEQGGNTSTPRSPYRTAAGSA
SPMQPNNTSKPKHRAAGGQTPERRASSDVHGQHTPGRSRMRQGYQGYNAEEEVAVPPFGA
WDEANAASGEKFTGIFNRVRDDKLSPNSSARQSSNANHGQENKVQQTCPCCIL

>Bd_KQJ99563
MAQNGIPAWGNWDSTDNTPYTQKFENVRRTKKTGVSSSPSDPRRSPEPPRKSPLHPSKYT
PEALDHSPKYQPHASKPEPDHPRPMASPLREPVPRRHANPLHQQHLDQGGYGSPYRATAG
AASPMQAGNAARSKHRSAGMQTPERRASSAVHGPLTPGRIGAKQGGRAYEVDDEVAVPPF
GGWDEGNAASGENYTGIFNRVRNDKLSPNSSAKQPSSYSGKQENKVQQTCPCCIL

>Bd_KQK17571
MQNRPSVPKFGTWDSDNARYTVYFEKIRENKGATAPPLHRPFNPNEDPPEEEDPAAPRLI
VTPAGSSSRPATSSGQRAAHHQQQPAHAHRRAGSSSSVPSDHGGGGRHHQSKFAPPPQYQ
PRPQAPAPGAHGHQPAAHGGDHGHHHRPTGHHAQHSSRQQQQQSSGSRARSASPRHSAQG
RQRPTAVPRFGVWDEQSAAAQGFTVQFDNVKRHREVARGRGVPDVPRRRMPSPESYAAAR
RRSSSRQHTPFVSKMFGCFLQHPTTRD

>Hv_HORVU5Hr1G121530.1
MQHQGVPKFGNWEDEGQGYSQYFENARMGKSPGRPVNQNDRNEGAAQAPSNDPPSVKASP
LRPGSEPGLRKNRDERRATREDDIRRHEAAARKSHAESPNHRYGDQANDDGAARKAGNER
SPIHPRQQARLANKGGVSSPIADRRGSAPTTPGRSKMRPTGRGDETPERGSAVPKFGDWD
EKDPSTGEGFTDIFEKVREEKQSGADTVGTSHAYTGRYNRGERYESSGCSCFSWFKK

>Hv_HORVU7Hr1G053300.1

MAKQQKNAHVPKFGNWDNDGNVPYTLYFDNARKGKGGKPMNPNDPVENPEAFSSSVVAPS
PNRSFDQARPAPALPPASPPPAHHHERRPSDGPAPAPPLSPYHRNAGGEPPRRGAGGGRA
GGYSVEQSPAPSPLHPYGQSKAEHSDGSSYGLVANANSVDRSRARAASRGNETPTRGSAV
PKFGDWDSNPASADGYTHIFNKVREEKSTQAKAPGFGKDNVAYGKGARQHDDGYVSSSRW
CFGWCK

>Hv_HORVU7Hr1G092240.2
RGWLDQGGYFAASASTAKGGSAIIRRRVKFKPAPLRPPPCLGRALPLRPPSPSSLRGKER
DGTQETGAERDQAQAPAAPSTPPRRMANRPSVPKFGTWDSDNVGYTVYFNKVRENKGATA
PPLQRPFNPNDPEEEAPRVIAPGSRPATSSGDRAAAQQNGQQHRRAGSSSSVATDRGGRV
AEKSKFAPPPQYHPRPQPAHHQPPAGHGGDNGHHRSPSGHGGDHGHHHHHLPAGAGHGGH
HKPQEARYKPAGARARSASPNHAPQRPTAVPKFGAWDEQSAAAQGYTVQFDRVKHDREAA
RRGAVPDLHRRRMPSPESYTAASRSRQHTPFYSKMFGCFLPHTTE

>Sb_OQU90612
MHQGVPKFGSWEDEGDHLYTQYFENARKGKSPGRSASQNDHSGDPEALSKDSPSAKASPL
RTGSDPVVQKPKDERRANREDDLRRHEAPARRPYAESATHKHGVNTSYDSAARKTGMERS
PLHPHHQARVVNKGGVSSPSWERRGSSEGHRGTAPTTPGRSKMRPSGRGDETPERGSAVP
KFGEWDEKDPSTGEGFTDIFNKVREEKQSGDAPVFTSDTGYNRSNQGRKYESSGCSCFSW
FRN

>Sb_KXG26087
MAHPEIPAFGDWETTGNTPYTQKFEDARKNKKTGIPTQPNDPRRNPEHPRKSPLHPTTYK
TDPQDQGPRNPPHRPRPGTDHQRHSDRPTHREPAPRRQANPQREQESNAGAPRSPYRTAV
GSASPMQPNNQSKPKHRLTGMQTPERRPSSEGHGQHTPGRSRMKQGGYEPEEEVAVPPFG
EWDDANAASGEKYTGIFNRVRDDRLSPTSSARQPSTTRSEENKVQQKCSCCIL

>Sb_KXG25553
MAQQKNAHVPKFGNWDNDGNVPYTLYFDNARKGKGAGGKMINPNDPAENPEAFSMAAPSP
AQTPPRHERRPSDAPPPAPVSPNPYAGSPYHHRHGGRGGGRAAAGGYSVEQSPVHPYSTS
ESAGYGLVANSVDRSRAKGGSRGNETPTRGSAVPKFGDWDSNPASAEGYTHIFNKVREEK
QTQAGKPAAYGKDGGARGNGAKQHHDDGYVSSKFSCFGWCKQ

>Sb_KXG20463
MAKRPTVPKFGAWDNDDVGYTVYFDKVRENKGATAPPLHRPHSLDDPEEGPMMRVPPPSS
SRPATAGGHREAPPSSRRHHGQGGHRRTESSSSVASDPGSAHQSKFAPPPQYYQRTSHGH
HRHHSGGHHHHQQQQQQQQQPPSSHGHGHGHGHRASHAHSRQQQHHHAAPGPRARSASPQ
SNAPRPSAVPRFGVWDEQSAESAAQGFTVVFENVKRHREVARSGVPAVPRVPSPPEGAAL
RRHHQKTPFVSKMFGCFLPTTAKG

Long two-NOI PAS

>Atr_ERM96932
MARNSGLGRTIATPNSISMYTQINHSHVPEFGNWESGENVPYTQYFENAVKTRGTGGKIM
NPNDPEENPEAFRYGTADSKSFDASPLHLASTSPKRRQYERHQSDVAERHGYPMYSPSRY
DSGARKVMHGSPIHGHTSYGRDMQSQGNSGSSPLRVRKSSRDGGHGLSPTSPVRFRSKGG
NSRDDDQLESVAAVPKFGAWDDRDPTSGDGFTVLFNKVKEERQIRAAKPPVIPTKPVVYP
TSYKKHNGSTRSSI

>Pp_Pp3c1_23970V3.2
MARPHVPKFGAWDAKGGNGGTYTAVFDHARAGKGGKLINPNDPAENDALAAQLYGGPLPT
QRNNDRSPSRGRQAYDTGRPEPPPRRPQQDRYGDGPSHSGERSSANPYASGAREAGRGAA
TPAMGRRSRNFSGGDEGSVLAPGTPKARLRTQGGRPEEPTKGGALPKFGDWDVKDPNAGE

GFTVIFQKLADEKKEGGPVQIPRLNPDHRLSHDEGHGKRSQYGASKVTKDTKHSRQPGCC
TIL

>Pp_Pp3c2_10900V3.1
MARPHVPKFGNWDANGGNGGAYTAVFDQARTGKGGKPINPNDPAENEALAAQIYGGPLPT
QRNNDRPPPRTRPAHVTEDAANRPRHERRSSREDLDVRRSNDSASRQPSSDYQGPARKPV
GGPGGRAEPPFRRPQPDMDGDGSSHNAERAPANPHANRMNARETGRGAASPAWERRGRNP
SGGDEGSVLGSGTLKSRPRPQGGRAEESGKGGALPKFGDWNEKDPNAGDGFTMIFQKLSN
EKREGGPVHIPRLNSDHQASHEDSLGKHSQLGVQKTSNANDQPKCCVIL

>Pp_Pp3c17_17040V3.1
MARPHVPKFGAWDAGAGNGSAYTAVFDQARTGKNGKPINPNDPAENEALAAQTYGGVPAH
RNNERRAHDENRPRHERRSSREDLDVRRSNDPPSRQPPHLDQPPARRPPGGVGGRGGAEA
PPRRPDRDVSNHSDRDGSNHNADRSPAHVHGNRLGARDAGRGAASPAWERRGRHPSGGDE
GSVTGSGAPKTRLRPAGPREEPPAKGGALPKFGAWDVKDPNAGDGFTMIFQKLSNEKKEG
GPVHIPRLNPEQQPSHEDSYVKHNQPGGPKGKKQSSRPDCGCTIL

>Pp_Pp3c14_15740V3.4
MARPNVPKFGAGTGAAYTAVFDQARTGKNGKPINPNDPAENVGLYGYMPPLPSQRNSERP
PRGHDENRPRHERRSSREDLDVRRSNDPPSRQPPYPGQAPARKPPGGVGARGGAEGPPTR
PDRDGSNHTDRDGSSHHTDRSPAHPHANRVGARENRRGAASPTWERRGRQPSGGDEGSLL
GSGAPKPRMRPAGPREEPPTKGGALPAFGAWDVKDPNAGDGFTMIFQKLSNEKKEGGPVH
IPKLNTEQLSSHEKSYDKHNQSNGSRDKKQSSQPVLLENNAVVRACLESCLNLYSF

>Pp_PAC:32981807.CDS.1
MAGHIPKFGDANHGNYSEVFGAPTRLIGRNGSGDVAKGLNNQGGNSRGGAPAGRGDLPGQ
EKDASADSRMRRGGPGSSEGSGNLRSAGGSGSYEKEVPPRRQGSGGKQGSSEREGQGLRK
SQDNILGSGRPRQVVGRGAPGRINDSSSKGGRTPRRDDDYAGAGHLPKFGDWDDNAGDSN
YTMMFQAAAEDRRGVPASHSRPEGEQRGGHYKPNNSKKTASSCWCFGA
